# Enhanced Grand Canonical Sampling of Occluded Water Sites Using Nonequilibrium Candidate Monte Carlo

**DOI:** 10.1021/acs.jctc.2c00823

**Published:** 2023-01-24

**Authors:** Oliver J. Melling, Marley L. Samways, Yunhui Ge, David L. Mobley, Jonathan W. Essex

**Affiliations:** † School of Chemistry, University of Southampton, SouthamptonSO17 1BJ, U.K.; ‡ Department of Pharmaceutical Sciences, University of California, Irvine, California92697, United States; § Department of Chemistry, University of California, Irvine, California92697, United States

## Abstract

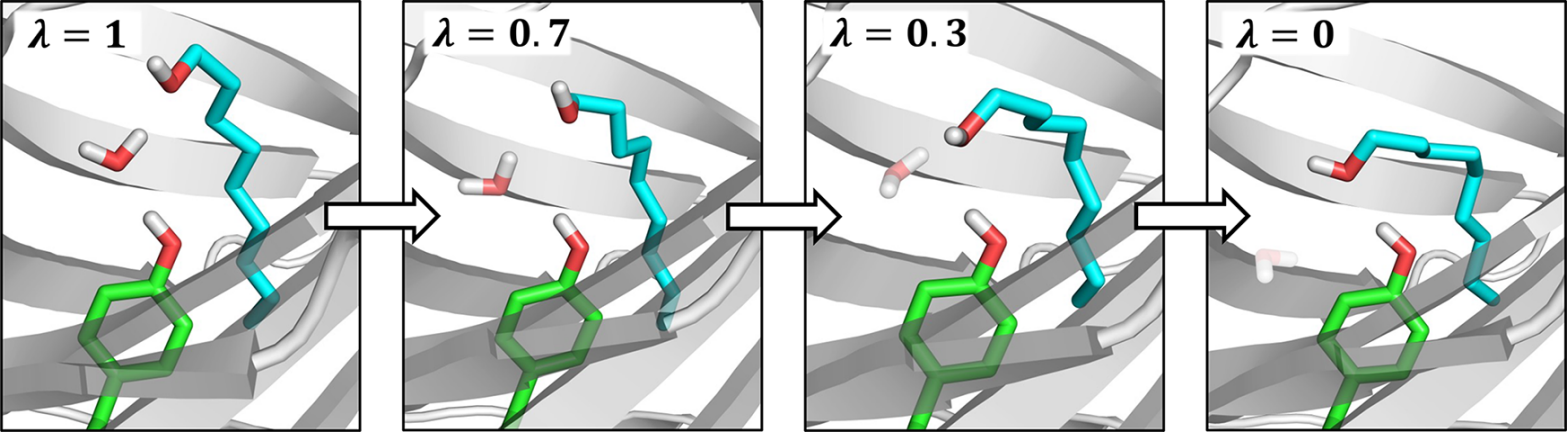

Water molecules play a key role in many biomolecular systems, particularly when bound at protein–ligand interfaces. However, molecular simulation studies on such systems are hampered by the relatively long time scales over which water exchange between a protein and solvent takes place. Grand canonical Monte Carlo (GCMC) is a simulation technique that avoids this issue by attempting the insertion and deletion of water molecules within a given structure. The approach is constrained by low acceptance probabilities for insertions in congested systems, however. To address this issue, here, we combine GCMC with nonequilibium candidate Monte Carlo (NCMC) to yield a method that we refer to as grand canonical nonequilibrium candidate Monte Carlo (GCNCMC), in which the water insertions and deletions are carried out in a gradual, nonequilibrium fashion. We validate this new approach by comparing GCNCMC and GCMC simulations of bulk water and three protein binding sites. We find that not only is the efficiency of the water sampling improved by GCNCMC but that it also results in increased sampling of ligand conformations in a protein binding site, revealing new water-mediated ligand-binding geometries that are not observed using alternative enhanced sampling techniques.

## 1 Introduction

Water molecules can have a significant impact on the affinity with which drug candidates bind to their targets.^1^ If a drug can be designed such that it displaces a water bound within a protein binding site, then the resulting gain in entropy can increase the drug’s affinity by up to 2 kcal mol^–1^.^2^ However, this is only the case if the modified ligand can replace the interactions that previously existed between the displaced water and the protein. As such, understanding the thermodynamics of bound waters, and the interplay between the entropic gain and enthalpic loss upon their displacement, is of great importance in drug discovery.^3^ Bound waters are prevalent in protein–ligand systems; previous studies have shown that over 85% of a data set of 392 high-resolution crystal structures contained at least one water molecule mediating the interaction between the ligand and the protein.^4^

Experimental methods suffer from a number of limitations when it comes to understanding the locations and thermodynamics of individual waters within protein structures. X-ray crystallography is predominantly used as the experimental method for determining water locations, although this gives rise to several issues. First, the conditions under which the crystals are formed are not necessarily comparable to physiological conditions, plus the protein may adopt different conformations in the solid phase to those adopted when in solution.^5^ Second, water is isoelectronic with several common ions and can therefore be either incorrectly assigned to a region of electron density or not assigned at all.^6^ Third, the assignment of water locations can be done in such a way that the overall unexplained density in the structure is reduced, meaning that while the model may contain fewer errors overall, the placement of individual water molecules is potentially less accurate.^7^ Computational methods therefore have a role to play in the understanding of both water locations within protein structures as well as their thermodynamics.^8^

Given the significance of protein-bound water sites^9,10^ and the difficulties associated with experimental investigations, a large amount of work has been dedicated to the development of computational methods in this field.^11^ This has been necessary as conventional molecular dynamics (MD) sampling methods can struggle to sample bound water molecules effectively, especially when the hydration site is buried within a cavity and occluded from bulk solvent.^12^ In such cases, kinetic barriers prevent the water from moving between the bound site and bulk within currently accessible simulation time scales, which are typically on the order of microseconds, compared to the millisecond time scales often required to observe water exchange between a solvent and buried sites.^13^ Given this poor sampling, the locations of any occluded bound waters typically need to be determined before a simulation and may remain unchanged throughout. As the occupancy and location of these waters can often be coupled to the conformations of the protein, the inability of conventional MD to rigorously sample these degrees of freedom can result in the generation of inaccurate ensembles and, in turn, errors in calculated properties, such as binding free energies.^14,15^

Grand Canonical Monte Carlo (GCMC) simulations have been in use for over 40 years, and their ability to sample the grand canonical ensemble in a theoretically rigorous fashion is accepted. As a result, they have been applied in a wide range of contexts such as investigating the binding of hydrogen to metal–organic frameworks and simulating the movement of ions through channels in membranes.^16−22^ Sampling the grand canonical ensemble requires the chemical potential (μ), volume (*V*), and temperature (*T*) to be held constant.^23−26^ Simulating at a constant chemical potential allows the number of particles in the system to fluctuate, which can be used to bypass kinetic barriers to the sampling of buried water molecules through randomly attempting their insertion and deletion within a user-defined region of interest such as a binding site.^12,18,20,27−30^ These attempted moves are accepted and rejected based on rigorous probabilities derived using the Metropolis-Hastings algorithm. The use of GCMC sampling has been found to significantly improve the accuracy of ligand binding free energy calculations, where displaced waters that are not expelled sufficiently quickly from the binding site can have a serious impact on the free energy results, when using conventional sampling methods.^12,29,31,32^ However, the acceptance rates for unbiased and instantaneous particle insertions and deletions in condensed phases are typically very low, with around 1 in every 10,000 moves attempting to insert/delete water molecules to/from a bulk water system being accepted.^33^ A number of enhanced sampling techniques have been developed to improve the acceptance rates of GCMC, including cavity biasing,^25^ continuous fractional component Monte Carlo,^34^ configurational biasing,^35^ and molecular exchange approaches.^36^ Here, we investigate the use of nonequilibrium switching to enhance the acceptance rates and, in turn, the efficiency of GCMC moves.

NCMC is an enhanced sampling technique designed to improve the acceptance of low-probability Monte Carlo moves between high-probability configurations by utilizing nonequilibrium switching processes.^37^ The method has been in use for over a decade, applied to a number of sampling problems, including changes in protonation states,^38,39^ ligand binding modes,^40,41^ rotation of restricted torsions,^42^ and fluctuations in salt concentration.^43^ NCMC is applied to a Monte Carlo move by breaking a large move proposal (such as a dihedral flip) into many smaller perturbations, interspersed with relaxation steps to allow the environment to respond to these changes. Whereas an instantaneous Monte Carlo move might result in a steric clash with the environment, which could cause an otherwise favorable proposal to be rejected, NCMC is intended to allow these clashes to be resolved before proposing a final state. In some cases, NCMC has been found to boost the acceptance rate by orders of magnitude over conventional Monte Carlo sampling.^40,43^

In this work, we present a combination of NCMC and GCMC. We refer to this new method as Grand Canonical Nonequilibrium Candidate Monte Carlo (GCNCMC). Rather than abruptly inserting or deleting a particle to/from the system, the particle is gradually coupled or decoupled in an alchemical fashion, governed by an alchemical coupling parameter (λ) where λ = 0 indicates a noninteracting particle and λ = 1 indicates a fully interacting particle. Performing these insertions and deletions gradually provides the opportunity for the environment to respond to the proposed change. As well as the effect of NCMC on the acceptance rate, we also consider the overall efficiency of the simulations—given that the use of NCMC introduces additional computational cost, it is important to assess if any increase in acceptance rate observed is worth the increased cost of the simulation. We test a number of different GCNCMC protocols on several protein systems of interest, and also on bulk water (which serves as a homogeneous test case to demonstrate proof of principle).

We find that GCNCMC offers significant advantage over GCMC in terms of acceptance rates and efficiency, but more importantly, it can also facilitate the sampling of new ligand conformations in the binding site.

## 2 Theory

### Grand Canonical Monte Carlo (GCMC)

To sample states with different numbers of particles, GCMC simulations employ Monte Carlo moves that attempt to either insert into or delete a particle from the system.^27,28^ The acceptance probabilities for these moves (when using the Adams formulation of GCMC^23,24^) are written as
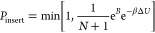
1
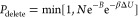
2where Δ*U* is the potential energy change associated with the proposed move, β is the inverse temperature, *N* is the number of particles before the attempted move, and *B* is the Adams parameter,^23,24^ defined as
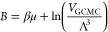
3where μ is the chemical potential, *V*_GCMC_ is the volume of the region in which GCMC moves are attempted, and Λ is the thermodynamic wavelength of a single particle. When water in equilibrium with bulk water is simulated, the corresponding Adams value (*B*_equil_) is determined as
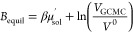
4where  is the excess chemical potential and *V*^0^ is the standard state volume of water. Here, these values are taken as −6.09 kcal mol^–1^ and 30.345 Å^3^, respectively, as determined in a previous work.^33^ The excess chemical potential is equivalent to the hydration free energy of a single water molecule. These parameters are dependent on the water model being used, which in this work is TIP3P. We have previously found the density distribution of bulk water to be highly sensitive to these parameters.^33^

The theory described up to this point explains a basic GCMC implementation, with no additional enhanced sampling used to increase the acceptance rate. Simulations are typically performed by alternating MD sampling on the whole system with batches of GCMC moves, and we refer to this as GCMC/MD.^33^

### Nonequilibrium Candidate Monte Carlo (NCMC)

An NCMC move is governed by a protocol (Λ_p_) that consists of a sequence of alternating perturbation (a change to the alchemical coupling parameter) and propagation (relaxation of the system) steps, which when applied to a set of system coordinates, yields a nonequilibrium trajectory (*X*) and a final proposed state (*x*_T_). To maintain detailed balance, there must also be a reverse protocol , which when applied to the proposed state after reversing the momenta (, where the tilde is used here to represent a state with reversed momenta), returns the system to the initial state, via a reverse trajectory .^37^ This results in the following, general NCMC acceptance ratio:
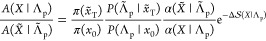
5where π(*x*_0_) is the equilibrium probability of state *x*_0_, *P*(Λ_p_|*x*_0_) is the probability of selecting protocol Λ_p_, given state *x*_0_, α(*X*|Λ_p_) is the cumulative probability of all the perturbation steps from protocol Λ_p_, and  is the conditional path action difference. The full derivation and explanation of the underlying theory can be found in the publication by Nilmeier et al.^37^ It should be noted that this acceptance ratio is highly generalized and is significantly simplified when applied to real problems.

### Grand Canonical Nonequilibrium Candidate Monte Carlo (GCNCMC)

Here, we show how the principles of NCMC can be applied to GCMC moves to allow the insertion or deletion of a particle to be performed gradually by making small incremental perturbations to the alchemical parameter, λ. At each value of λ, including λ = 0 and λ = 1, a short amount of MD sampling is performed, referred to as propagation or relaxation. A number of simplifications can be made to the general NCMC acceptance ratio in eq 5, such that  because of the deterministic nature of the perturbation steps, to generate the acceptance probabilities for insertion and deletion moves shown in eqs 6 and 7. A full derivation of these probabilities is available in the Supporting Information.
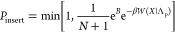
6
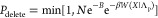
7Here,  is the work done by the nonequilibrium process. These equations are very similar to eqs 1 and 2, except that the potential energy change has been replaced by the nonequilibrium work. While the work done contains contributions from both the protocol work (the sum of potential energy changes caused by the perturbation) and the shadow work (additional work introduced by the integrator error during the propagation steps), here the shadow work is neglected. This is a common approximation,^40−43^ given that the BAOAB Langevin integrator^44,45^ used in this work has been found to preserve the equilibrium distribution with high fidelity.^46^

It should be noted that some additional considerations arise when the insertions and deletions of particles are attempted only within a subset of the total system volume, as is the case in this work where moves are only attempted within a sphere placed around a region of interest. First, if the water that is subjected to nonequilibrium switching lies outside the sphere at the end of an insertion move, the move is automatically rejected as it becomes nonreversible. Second, the  and *N* terms in eqs 6 and 7 must be adjusted to account for the fact that the number of waters in the sphere may change during the nonequilibrium protocol because of diffusion during the MD propagation steps and are, therefore, replaced with the following:
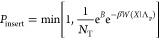
8
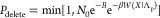
9where *N*_0_ is the number of particles in the GCMC sphere in the initial state and *N*_T_ is the corresponding number for the proposed state.

GCNCMC moves are implemented as described here in version 1.1.0 onward of the *grand* module.^33^

## 3 Methods

### GCNCMC/MD Implementation

In this work we refer to NCMC protocols in terms of their switching time, which is the total length of all propagation steps in each NCMC move (typically between 5 and 25 ps), the number of steps of propagation between each perturbation of the alchemical coupling parameter (*n*_prop_), and the total number of equally spaced perturbation steps between λ = 0 and λ = 1 inclusive (*n*_pert_). A move consists of alternating perturbations and propagations, with the latter making up the first and last part of each move (to ensure symmetry of the forward and reverse protocols). For example, if the desired switching time is 10 ps, then this could be achieved through an *n*_prop_ = 10 and an *n*_pert_ = 499 (assuming a time step of 2 fs). For a fixed switching time, if the *n*_prop_ value is increased, the *n*_pert_ value must be decreased, resulting in fewer, larger perturbation steps that are each separated by a longer period of propagation. The three parameters are linked by the following equation:
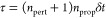
10where τ is the switching time, *n*_pert_ is the number of perturbations, *n*_prop_ is the number of propagation steps between each perturbation, and δ*t* is the time step. To avoid ambiguity, a brief list of definitions of these terms is provided:

- switching time (τ): the total length of an NCMC move—the sum of all the propagation steps
- perturbation: a change to the alchemical parameter, λ
- relaxation/propagation: some sampling of the whole system before and after each perturbation during an NCMC move (in this work we use MD sampling)

As is common in simulation algorithms that involve the insertion or deletion of atoms, such as relative binding free energy calculations, one needs to ensure that the unphysical states created do not result in high energies that cause numerical instabilities. In *grand*, a soft-core potential is employed to ensure that energies arising from Lennard-Jones interactions do not approach infinity when two particles are in very close proximity (as may be the case at the beginning of an insertion move).^47^ The soft-core potential used here is of the form^47^
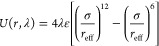
11where *r* represents the distance between two interacting atoms, λ is the alchemical coupling parameter, and ϵ and σ are the Lennard-Jones parameters. The effective distance (*r*_eff_) is calculated as
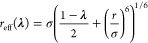
12Additionally, to ensure that strong electrostatic interactions are not left “bare” at small λ values, again potentially resulting in excessively high energies and forces, the Lennard-Jones and electrostatic interactions are scaled separately. Between λ = 0 and λ = 0.5 the Lennard-Jones parameters are scaled from noninteracting to fully interacting, and between λ = 0.5 and λ = 1 the electrostatic interactions are similarly scaled.

To summarize, the complete procedure of a single GCNCMC move begins by selecting, with equal probability, whether an insertion or deletion move is to be attempted. For an insertion move, a noninteracting water is placed at a random location within the desired spherical region in the system with a random orientation. For a deletion move, a water within the sphere is selected at random. The nonbonded interactions of the water are then gradually scaled over a series of perturbations, separated by periods of relaxation, with the direction of the scaling depending on whether the water is being inserted or deleted from the system. An acceptance test is then performed on the nonequilibrium work accumulated over the course of the move. If the test is passed, then the simulation continues from the final configuration of the GCNCMC move. If the test is failed, then the simulation restarts from the configuration immediately prior to the beginning of the GCNCMC move.

To achieve a balance of enhanced water sampling, while also continuing to sample the system as a whole, a typical simulation involves iterations of a single GCNCMC move followed by a short burst of traditional MD sampling (often around 5–10 ps in length). As with GCMC/MD, we refer to this simulation method of alternating GCNCMC moves with MD sampling as GCNCMC/MD.

### Water Hopping

This work makes comparisons to both the existing GCMC/MD method (as implemented in *grand*) as well as another enhanced water sampling method known as *water hopping*,^48−50^ as implemented in the *BLUES* module.^40^ The water-hopping method keeps the total particle number constant and generates trial states through translation of water molecules within the system. The translation is performed through a similar NCMC switching process to the grand canonical methods whereby a water is gradually decoupled from the system before being translated and then recoupled. A sphere is employed within which the water translation move takes place. The sphere must include both the region of interest as well as some bulk solvent to provide waters for translation. As such, the sphere required for water hopping is typically much larger than that required for the grand canonical methods, which can result in moves being accepted that transfer waters between different regions of bulk rather than between bulk and a binding site or other region of interest in the protein.

### Test Systems

Four test systems were used to both validate our GCNCMC implementation and assess its efficacy: bulk water, heat shock protein 90 (HSP90, PDB code: 5J64), the major urinary protein (MUP-I, PDB code: 1ZNK), and trypsin (PDB code: 5MOQ). All three proteins had a bound ligand. The structures for all the protein systems were obtained using X-ray crystallography with the exception of the trypsin system whose structure was generated using a combination of X-ray and neutron diffraction data. The neutron diffraction data were obtained at room temperature.

GCNCMC/MD, GCMC/MD, and water-hopping simulations were all carried out by alternating MC moves with MD sampling. For the two NCMC methods, NCMC moves were separated by either 5 ps of MD for the protein test cases or 10 ps of MD for the bulk water system. For GCMC/MD, GCMC moves were run in batches of 20, with the batches separated by 4 ps of MD sampling. The bulk water simulations are an exception as a number of different protocols were tested, as discussed below.

For HSP90, a total of 12 independent repeats were performed using each of the three methods, and for trypsin, 8 GCNCMC/MD repeats and 6 GCMC/MD repeats were run.

For the MUP-I system, 12 GCNCMC/MD, 8 GCMC/MD, and 8 water-hopping repeats were carried out. Six repeats of 100 ns each using plain MD were also performed to act as a control with no enhanced sampling. Simulations were also performed with positional restraints applied to the protein and ligand to hold the system in four different conformations identified. For each of these four ligand conformations, 8 repeats of GCNCMC/MD, GCMC/MD, and water hopping were carried out. A force constant of 10 kcal Å^–2^ mol^–1^ was applied to all nonsolvent heavy atoms as the restraint in these cases.

### Analysis Methods

Where clustering analyses were performed, this was done using average-linkage hierarchical clustering (as implemented in *SciPy*(51)) of the simulated water oxygen atom positions with a distance cutoff of 2.4 Å, as described in a previous work.^33^ This allows the location of the simulated waters to be compared to that of the waters in the crystal structures. We use a threshold of 1.4 Å (the van der Waals radius of a water molecule) to determine whether the location of a cluster is in agreement with that of a crystallographic water.

Electron density calculations were performed using the *LUNUS* software,^52^ which allows mean structure factors to be computed from the states generated during an MD simulation, from which electron density maps can be generated. The calculated maps were compared to the experimental 2*F*_o_-*F*_c_ maps with a contour level of 1.5σ used for the experimental maps and 3σ for the calculated maps. The approach of calculating electron density maps from simulation data has been used previously by Ge et al. in a similar study and is explained in greater detail in their publication.^50^ This allows for a secondary comparison between experimental and simulation data, in addition to the clustering analysis described above. Electron density calculations were not performed for the trypsin system, owing to the experimental structure being obtained via neutron scattering, or on the MUP-I structure, given that many of the conformations generated were distinct from the crystal conformation and the results would not have provided any further insight.

Throughout this work, force evaluations are used as a way of comparing efficiency across different methods, given that they are the most expensive calculation in a typical MD algorithm. We use the term “force evaluations” to refer to any calculation that requires the interatomic distances to be calculated, which includes both energy and force calculations. Force evaluations are calculated as detailed in Bergazin et al.^49^ with each MD step (whether normal MD or NCMC propagation) and each GCMC move counting as a single force evaluation.

### System Setup

The AmberTools tleap software^53^ was used to generate and solvate the simulation boxes and add ions to neutralize the systems. Where necessary, the H++ web server^54−56^ was used to protonate the systems, with more details on the protonation states provided below. The details of each system are provided in Table 1, including the radius and atoms used to define the GCMC sphere. For the water-hopping simulations, sphere radii of 1.5 and 2.0 nm were used for HSP90 and MUP-I, respectively. For HSP90, the sphere was centered on the C4 atom of the ligand, and for MUP-I it was centered on the C9 atom of the ligand, where the atoms are labeled as per the associated PDB file\z.

**Table 1:** Four Test Systems Used in This Work^a^

system	system volume (nm^3^)	no. atoms	sphere radius (Å)	sphere atoms (C_α_)
water box	64.02	6282	none	none
HSP90	223.90	22446	6	Leu48, Gly97
trypsin	226.50	22681	6	Gly226, Ala221
MUP-I	210.37	21170	6	Lys55, Leu117

a Listed with the volume of each system, the number of atoms after equilibration (n.b. this may change during the simulation), the radius used to define the spherical region of interest for water insertion and deletion moves, and the backbone C_α_ atoms whose midpoint was used to define the center of the GCMC sphere (residue IDs as per the crystal structures).

The HSP90 and MUP-I crystal structures had no missing residues, and once protons were added to the protein, all Asp and Glu residues were negatively charged, whereas all Lys and Arg residues were positively charged. For HSP90, all histidine residues were protonated on the δ-nitrogen, and eight sodium ions were added to neutralize the system. For MUP-1, His20 and His104 were protonated on the ε-nitrogen, and His46, His57, and His141 were protonated on the δ-nitrogen. A total of 13 sodium ions were added to neutralize the system.

Some residues in the trypsin structure had missing heavy atoms, as detailed in the PDB file, which were modeled using MODELLER.^57^ Where protons were present in the crystal structure, they were retained during the system setup, and any missing protons were modeled as described above. The protonation states of all residue side chains were retained from the crystal structure. Where residue side chains were resolved in multiple conformations in the crystal structure, the conformation with the highest occupancy was used in the starting simulation structure.

### Simulation Details

All simulations were performed in OpenMM 7.3.1^58,59^ with the proteins and waters modeled with the AMBER ff14sb and TIP3P force field parameters, respectively.^60,61^ Joung-Cheatham parameters were used to model the neutralizing ions.^62,63^ The ligands were modeled with the general AMBER force field^64^ (GAFF) with AM1-BCC charges.^65,66^ For the benzamidine ligand in the trypsin system, an atom type of “nh” was incorrectly assigned to the nitrogen atoms, so these were manually changed to an atom type of “na”. Lennard-Jones interactions were switched to zero between 1.0 and 1.2 nm, where the Particle Mesh Ewald^67^ (PME) method was used to calculate the long-range electrostatic contribution. The SETTLE algorithm^68^ was used to constrain the bonds in water molecules, and the SHAKE algorithm^69,70^ was used for all other hydrogen-containing bonds. All simulations were run at a temperature of 298 K. The Langevin BAOAB integrator^44,45^ was used with a time step of 2 fs and a collision frequency of 1.0 ps^–1^. The NPT sampling performed during the equilibration used a Monte Carlo barostat to maintain the pressure at a value of 1 bar.

**Figure 1:**
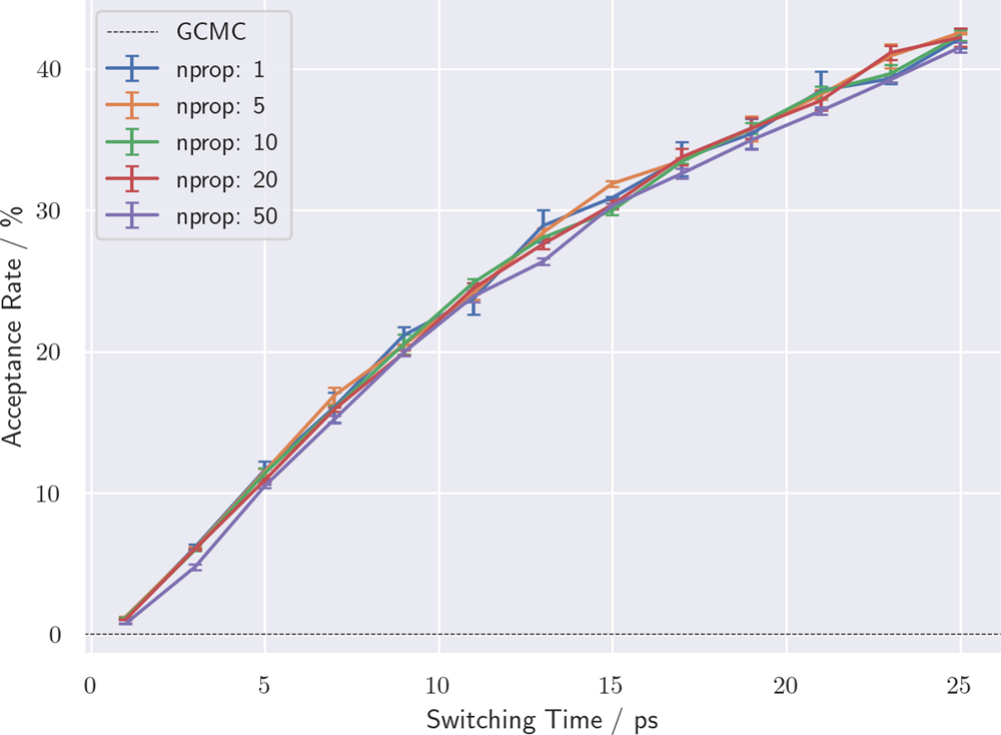
Acceptance rates of NCMC-enhanced GCMC moves within bulk water over a range of switching times from 1 to 25 ps. The *n*_prop_ parameter indicates the number of MD steps between each perturbation step. The error bars show the standard error of the mean accumulateed over three repeat simulations. The dashed line shows the acceptance rate for GCMC/MD moves.

**Figure 2:**
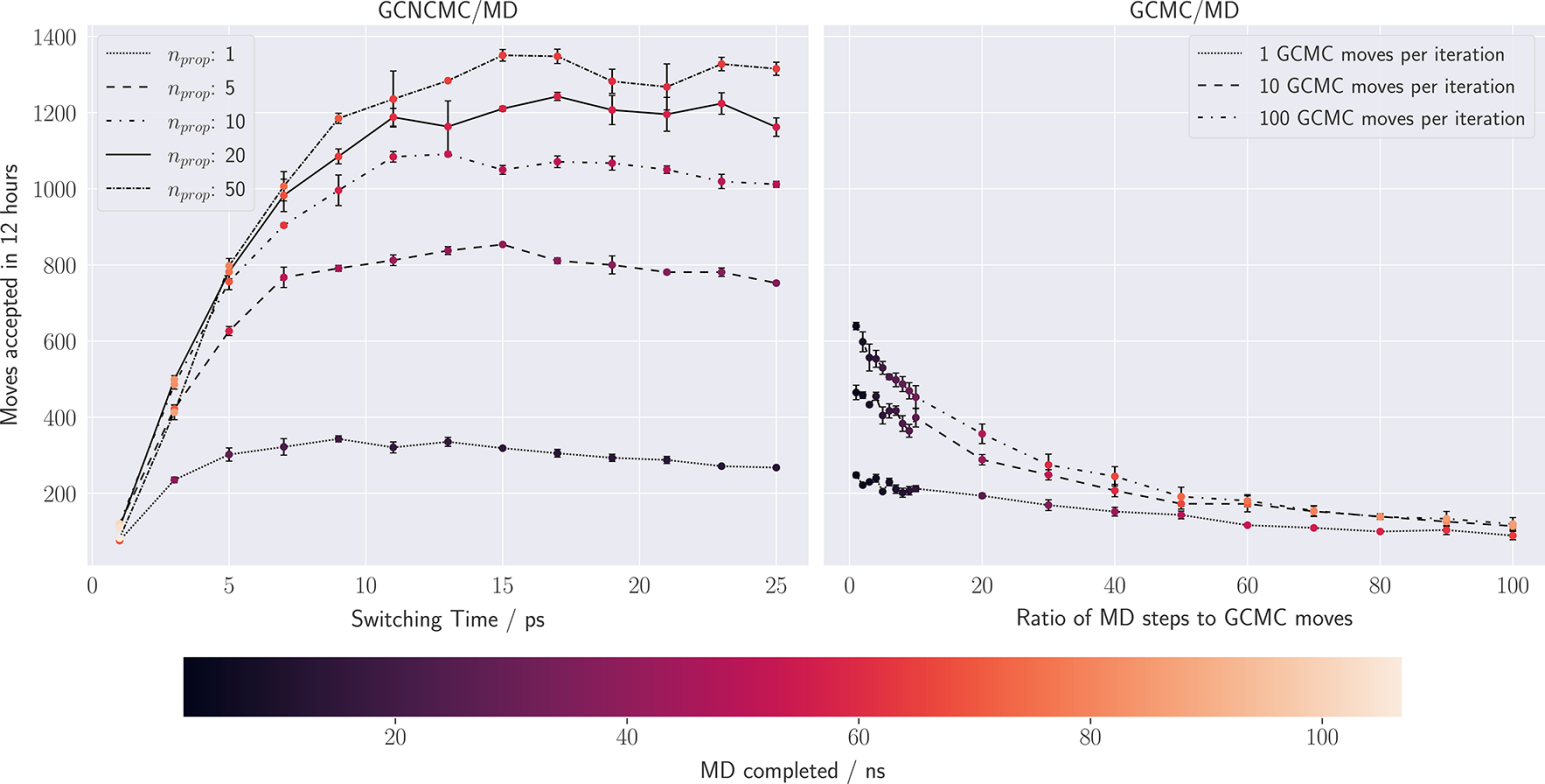
A comparison of the efficiency of GCNCMC/MD (left) and GCMC/MD (right) on a bulk water system. Efficiency is measured as the absolute number of moves accepted in 12 h of wall time. Points are colored based on the amount of MD completed during the simulation. MD performed during accepted GCNCMC moves is included in this calculation. The GCNCMC/MD data are grouped based on the number of MD steps between each perturbation during the NCMC move and plotted against the switching time of a single move proposal. The GCMC/MD data are grouped by the number of moves per iteration and plotted against the ratio of the MD steps to GCMC moves.

A range of protocols were used for the NCMC simulations. These generally had switching times between 5 and 10 ps, and all had an *n*_prop_ value of either 20 or 50. A full list of the protocols used for each system can be found in Table S1 (Supporting Information). The switching times were chosen based on the results shown in Figures 1 and 2 and an analysis performed previously of different switching times on protein systems. Where these data showed no clear optimal switching time, a value of between 5 and 15 ps, and more often 7 and 11 ps, appeared to be a reasonable choice for protein systems.

Frames were written out at the end of each iteration, with an iteration being a single block of MC and MD sampling. For the MD simulations of MUP-I, frames were written out every 10 ps.

### Equilibration Protocol

The equilibration of the protein–ligand systems was performed using a combination of MD sampling and instantaneous GCMC moves as implemented in *grand* version 1.1.0.^33^ An initial 10,000 GCMC moves followed by 100 iterations of 1000 GCMC moves and 10 fs MD allowed any structurally important water sites to be hydrated. Then 500 ps of MD in the NPT ensemble was used to ensure the system volume was correctly equilibrated, before a final 500 iterations of 1 ps MD and 200 GCMC moves finished the equilibration.

## 4 Results and Discussion

### Bulk Water Acceptance Rate and Efficiency

A comparison of GCNCMC/MD and GCMC/MD on a water box (*B*_equil_ = −2.630) showed that enhancing the sampling with NCMC resulted in an increase in both acceptance rate and efficiency. The average acceptance rate with GCMC/MD was 0.028%, whereas with GCNCMC/MD, acceptance rates of up to 40% were observed, as shown in Figure 1. The acceptance rates appear largely independent of the spacing between perturbations (*n*_prop_), although at the largest spacing, with 50 propagation steps between perturbations (and hence fewer, larger perturbations), the acceptance rates begin to drop below the trend line—the perturbations become large enough that even the longer propagation time is not sufficient to allow the system to relax. It should be noted that where these acceptance rates demonstrate a huge improvement on GCMC/MD, such a large improvement is unlikely to be observed in a protein–ligand system, owing to there typically being a few locations where waters bind, unlike the homogeneity of bulk water where insertions and deletions anywhere in the box have a reasonable chance of acceptance, given sufficient relaxation.

An increase in acceptance rate does not necessarily lead to an increase in efficiency, however, as the additional time required to generate the trial states when using NCMC needs to be considered. As such, an analysis of the efficiency was performed, here defined as the number of moves accepted in 12 h of wall time. All simulations were carried out on identical hardware (GPU: GTX1080, CPU: Intel Xeon E5-2680 v4). Figure 2 shows the relative efficiencies of GCNCMC/MD and GCMC/MD. The GCNCMC/MD simulations were run by alternating single GCNCMC moves with 10 ps MD as an example of a protocol that allows good sampling of both the system and waters. The GCMC/MD simulations were run as cycles of a block of GCMC moves followed by a short period of MD. Both the absolute number of GCMC moves and the ratio of MD sampling to GCMC moves were varied.

The results of the efficiency analysis shown in Figure 2 are clear; despite the greater computational time required to generate trial states, the GCNCMC/MD method remains more efficient. The efficiency is dependent on both the switching time and the spacing between perturbations. As the switching time increases, the efficiency rises initially, owing to the increase in acceptance rates, but it reaches a peak where the longer time taken to generate the trial states is no longer compensated by the increase in acceptance rate. Were it not for the consistent 10 ps MD between GCNCMC moves across all switching times, the decline after the peak would be steeper. Figure S1 (Supporting Information) shows additional data points at a switching time of 50 ps, demonstrating the decline after the plateau of around 15–20 ps.

As the length of the propagation between perturbation steps decreases, the efficiency also decreases as a result of having to pause the simulation more frequently to make the necessary alchemical changes (which are carried out off-GPU, as implemented in *grand*). The most efficient protocol was a switching time of 15 ps with 50 MD steps between each perturbation (149 total perturbations), which on average accepted 1351 ± 15 moves and sampled 64.8 ± 0.8 ns MD during 12 h of wall time.

The efficiency of the methods, independent of our implementation, was also measured by comparing the number of moves accepted within 1 × 10^6^ force evaluations. The comparison is shown in Figure S2. The results show that GCMC/MD protocols perform slightly better than GCNCMC/MD, once both the accepted moves and MD sampling is considered, with a ratio of about 5–10 MD steps per GCMC move being optimal. There is also no longer a dependence of the efficiency on the number of perturbations and their spacing—suggesting this is a purely an artifact of our particular implementation.

### Protein Test Case 1: HSP90

**Figure 3:**
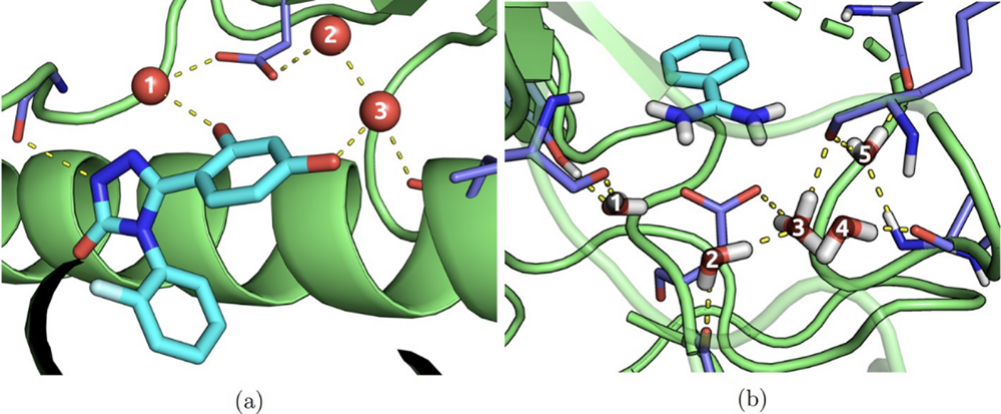
Locations of the bound waters in the crystal structures of HSP90 [(a) PDB code: 5J64) and trypsin ([b] PDB code: 5MOQ). The labels given in these figures are used to refer to the water sites in the main text. In the case of the trypsin structure, both X-ray and neutron scattering data were used to generate the final configuration, hence the presence of hydrogen/deuterium atoms (although the apolar hydrogens are not shown, for clarity).^71^ Where more than two hydrogen atoms are shown for a single water (as per water 3), this indicates that the hydrogen atom occupancy was split across three sites.

The crystal structure of HSP90 contains three water molecules that mediate the interactions between the ligand and the protein, as shown in Figure 3a. Preliminary results suggest these waters are tightly bound, and as such we expected that the three water sampling methods would generate ensembles in which the three sites were highly occupied.

The GCNCMC/MD results showed that, over the course of the finite simulation, water 1 had 100% occupancy as at no point was a move attempting to decouple it from the system accepted. Water 3 was fully occupied in 10 of the 12 repeats. In the two cases where a deletion move was accepted, the site remained vacant for five iterations of MD + GCNCMC (0.125% of all states) as the water on crystal site 2 moved to fill the gap—suggesting a greater stability of hydrating site 3 over site 2. The occupancy of the water 2 site was 99.5 ± 0.2%, with half of the simulations showing this site to be fully occupied. The errors associated with the occupancies are the standard errors calculated over the simulation repeats.

Neither the GCMC/MD nor the water-hopping methods accepted any moves, which affected the water network within the binding site, and as such all three bound waters were present in every state generated by these methods.

**Figure 4:**
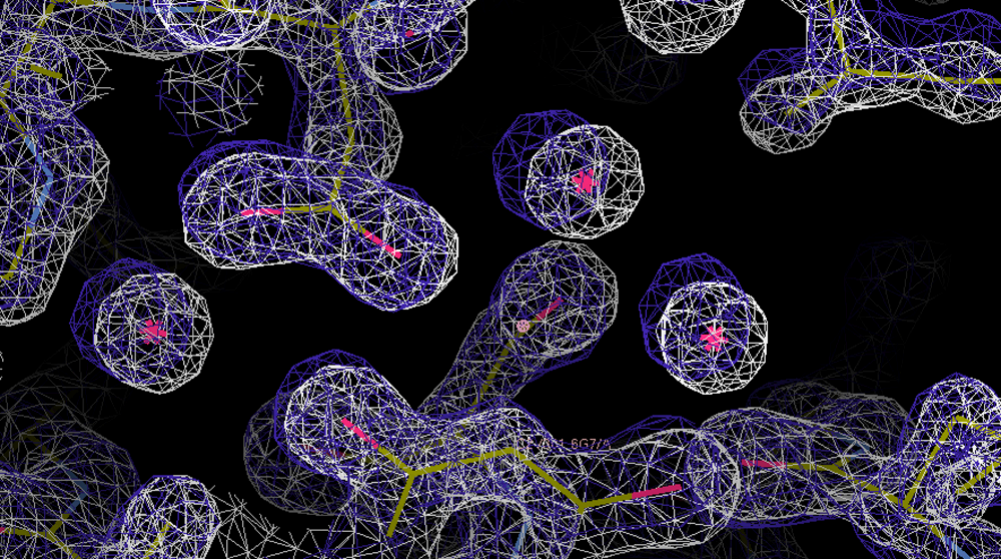
Experimental and calculated electron density maps are shown as white and purple meshes, respectively. The three crystal waters are shown by the pink asterisks, each surrounded by a region of electron density in both maps.

The electron density analysis was performed on all the simulations, confirming the results of the clustering analysis. An example of the electron density map generated from one of the GCNCMC/MD repeats is shown in Figure 4, where the overlap between the calculated and experimental electron densities is clear.

### Protein Test Case 2: Trypsin

**Figure 5:**
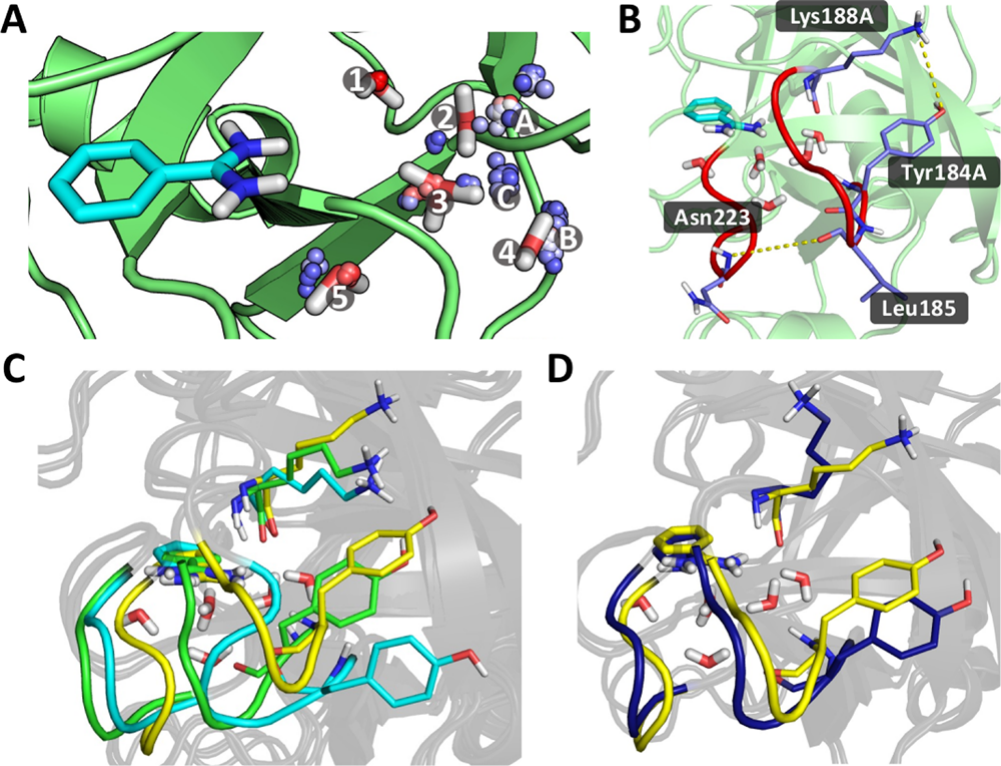
(a) Results of the clustering analyses performed on all GCNCMC/MD and GCMC/MD simulations. The crystal waters are shown as sticks, and the spheres represent the locations of the clusters with occupancies >20%. The occupancy of the cluster increases as its color changes from blue to white to red. Numbers are used to label the crystal waters and letters to label some of the hydration sites identified by the simulations. (b) Loops at the back of the channel that control the amount of diffusion with bulk solvent are shown in red. The dashed lines between the labeled residues show the distances used to measure how close the loops were to each other (Asn223-Leu185) and the extent to which the gap was blocked by the residue side chains (Lys188A-Tyr184A). (c) Example snapshots from the simulations. The yellow structure shows the most common conformation of the loops and the residues. The green structure shows an example of the gap between the loops closing but with the Tyr184A and the Lys188A blocking the pocket, and the cyan structure again shows the two loops close together but with the Tyr184A residue having shifted, allowing diffusion of waters into the channel. (d) Example snapshots from the simulations. The yellow structure is the same as that in (c), whereas the blue structure shows an example where the gap between the loops is mostly unchanged, but the Lys188A residue has adopted a conformation such that there is now space for waters to diffuse in and out of the channel.

The crystal structure of trypsin identified five bound waters located in the channel behind the binding site, as shown in Figure 3b. The locations and occupancies of all clusters with at least 20% occupancy are shown in Figure 5a. There was generally good agreement between the GCNCMC/MD and GCMC/MD simulations.

The simulation results were not always in agreement with the crystal structure, however; whereas the locations of waters 1 and 5 were consistent between simulation and experimental results, the locations differed for waters 2–4. For water 1, all GCNCMC/MD repeats showed a cluster within at most 0.3 Å of the crystal site (after alignment of the protein C_α_ atoms) with an average occupancy of 99.4 ± 0.3%, while the GCMC/MD repeats also all had a cluster within 0.3 Å of the crystal site, again with an average occupancy of 99.4 ± 0.3%.

The results for water 5 were slightly more varied across repeats but still showed good agreement between the two methods. The GCNCMC/MD simulations all showed a cluster within at most 0.3 Å of the oxygen atom of the crystal site with an average occupancy of 91 ± 2%, and the GCMC/MD simulations also all showed a cluster within 0.3 Å of the oxygen of the crystal water with an average occupancy of 92 ± 1%.

Water 3 was identified with some consistency by both methods, although this was typically on a site slightly offset from the location of the crystal water: shifted by approximately 0.6 Å toward the ligand. The approximate occupancies of this site were 76 ± 3 for GCNCMC/MD and 79 ± 2% for GCMC/MD. The cause of this offset is possibly due to the diffusion with bulk water observed at the back of the channel, creating an extra hydration site (cluster C) between crystal waters 3 and 4, as discussed below.

The cluster locations observed toward the back of the channel around crystal waters 2 and 4 were less well-defined. Both simulation methods identified hydration sites offset from crystal waters 2 and 4 toward the back of the pocket, as shown by clusters A and B in Figure 5a, which were found about 1.4 and 0.8 Å from crystal waters 2 and 4, respectively.

Structural analysis showed there to be a rearrangement of the loops at the back of the channel, which influences the extent to which water molecules can diffuse between the channel and bulk solvent. The loops in question are shown in red in Figure 5b. When the loops are separated by approximately 6 Å (as measured by the distance between the backbone nitrogen of Asn223 and the backbone carbonyl oxygen of Leu185) and the Tyr184A and Lys188A residues are in the conformation as shown in Figure 5b, there is minimal diffusion between the bound waters and bulk solvent, with the number of waters present in the channel fluctuating typically between 4 and 7.

A widening of the gap between the two loops leads to sufficient space being created for waters to diffuse in and out of the channel. A shortening of the distance between the two loops has a similar effect, although with the gap now being on the other side of the loop on the right. However, in this latter case, water translation is observed only if the Tyr184A and Lys188A side chains are not blocking the gap (Figure 5c, cyan). If these two residues are arranged as shown by the green structure in Figure 5c, then diffusion is less common. It is also possible for diffusion to occur if the loops are unmoved, but the Tyr and Lys side chains change conformations such that the gap between them increases, as shown by the blue structure in Figure 5d. In all these examples, where increased diffusion of waters between the bound sites and bulk solvent is observed, up to nine waters were observed in the GCNCMC sphere region.

The multiple protein conformations described above are a plausible explanation for the additional hydration sites identified at the back of the channel by these simulations. This in turn explains why crystal waters 2, 3, and 4 are identified with less accuracy and precision compared to crystal waters 1 and 5, given that the additional waters are likely to disrupt the water network present in the crystal structure.

### Protein Test Case 3: MUP-I

**Figure 6:**
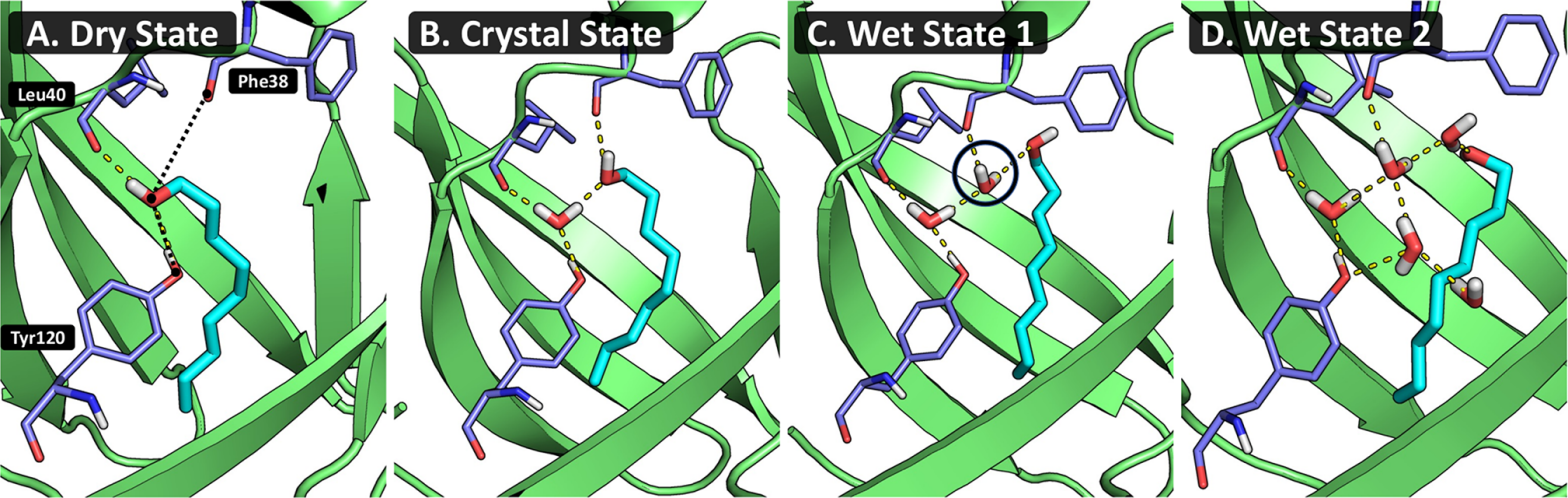
Four dominant binding poses of the MUP-I system. The ligand is shown in cyan, and the residues Tyr120, Leu40, and Phe38 are shown in purple. The residues are labeled in the leftmost figure, and the distances used for ligand conformation analysis, in Figure 7, are shown by dotted black lines. Dashed yellow lines show hydrogen bond interactions. The number of waters associated with each conformation increases from left to right. The second image (B) shows the crystal structure of the system. For ease of reference, the states are described from left to right as (A) dry state, (B) crystal state, (C) wet state 1, and (D) wet state 2.

**Figure 7:**
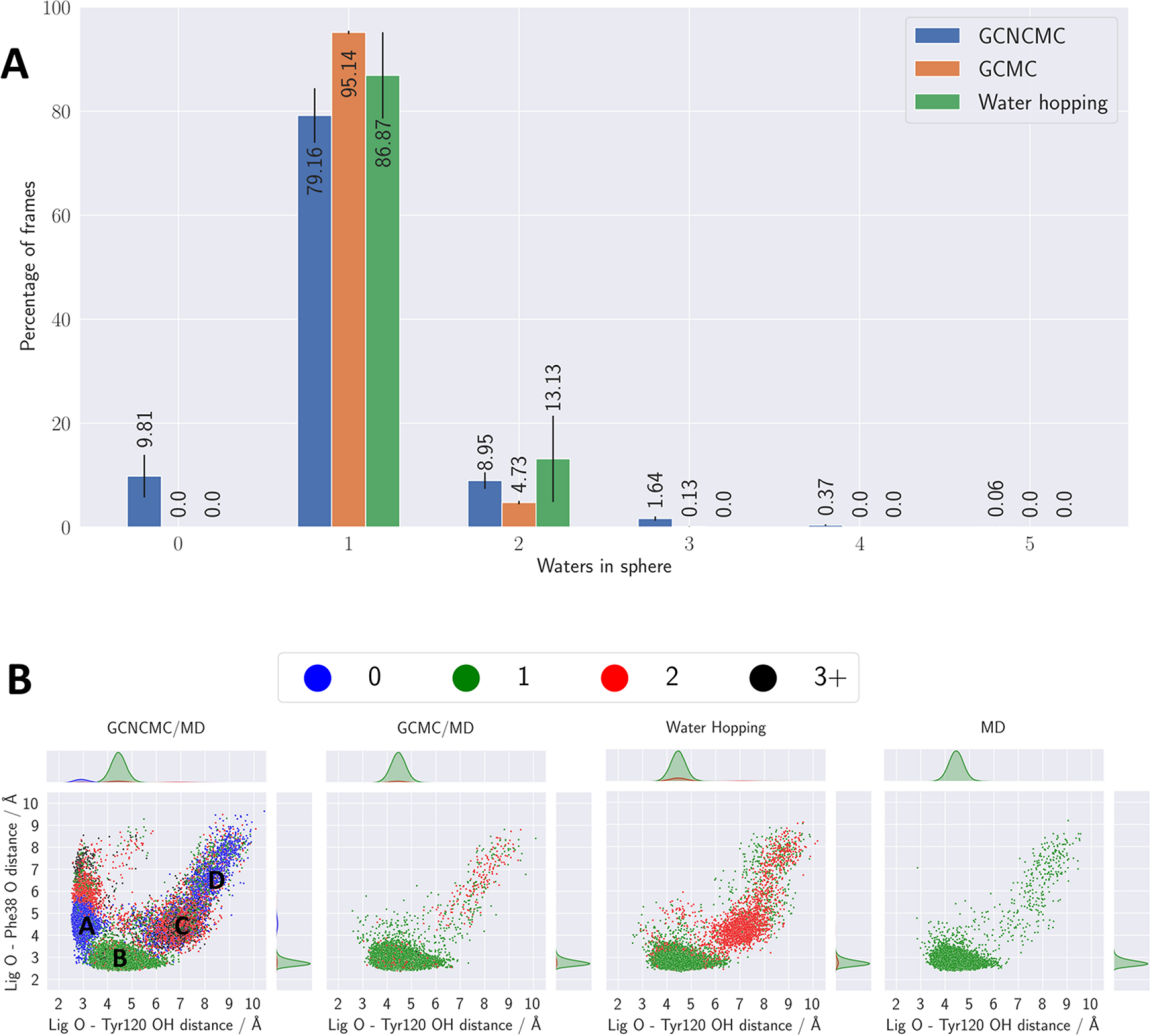
(a) The distribution of the number of waters observed within the MUP-I binding site across the three different water-enhanced sampling methods. Error bars show the standard error of the mean over the repeats. (b) The different conformations adopted by the ligand across the four different methods. The ligand conformational space is described by the distance from the oxygen of the ligand to both the backbone carbonyl oxygen of Phe38 and to the hydroxy oxygen of Tyr120, as shown in Figure 6. The points are colored based on the number of waters present in the binding site. The letters shown on the GCNCMC/MD plot indicate the four main ligand conformations in the same order, as discussed in the text [(A) dry state; (B) crystal state; (C) wet state 1; (D) wet state 2.

The crystal structure of MUP-I has one water present in the binding site, which mediates the interaction between the hydroxy groups of the ligand and residue Tyr120, as shown in Figure 6. This system was chosen as it had previously been used as a negative control case because of its expected low water occupancy within the binding region.^27^Figure 7 shows the distributions of the number of waters present in the binding site observed for MUP-I across the three different water-sampling methods. There is a clear lack of agreement across the three methods. The GCMC/MD results appear to be converged, with about 95% of the states containing just one water and 5% with two waters within the binding site. However, GCNCMC/MD and water hopping show broader distributions. Although the water-hopping method only generates states with either one or two waters present in the sphere, the GCNCMC/MD method generates states with anywhere between 0 and 5 waters present. Most notably, almost 10% of the states sampled using GCNCMC/MD contain no waters within the binding site—this is not observed at all with the other two methods. This is a far more significant difference between methods than that observed with the other test systems and warrants further investigation to ensure this is not the result of an error. If genuine, it suggests that enhancing the grand canonical sampling with NCMC is potentially providing a greater benefit than simply improving the efficiency of water sampling.

Structural analysis of the ensembles generated shows not only that multiple distinct conformations are adopted by the ligand (particularly in the GCNCMC/MD simulations) but also that a clear coupling between these conformations and the number of waters present in the binding site. We use the term “binding pose” to refer to a ligand conformation and the associated water network. Although the crystal structure binding pose is by far the most populated, three other binding poses also make notable contributions to the ensemble, and all are described below. All four conformations are depicted in Figure 6 (ligand shown in cyan).

- (A) Dry State: The ligand hydroxy group occupies the site where the water is observed in the crystal structure. No waters are present within the binding site.
- (B) Crystal State: The dominant conformation observed across all simulations. The single water present within the binding site bridges the interaction between the ligand hydroxy group and residue Tyr120.
- (C) Wet State 1: The ligand moves toward the back of the pocket, often coupled with a water occupying the site of the ligand hydroxy group in the crystal structure. When in this conformation, two water molecules are typically observed within the binding site.
- (D) Wet State 2: The ligand continues to move further toward the back of the pocket, again, often coupled with a water occupying the location of the ligand hydroxy group in the previous conformation. It is this binding pose that leads to states with up to five waters in the binding site, owing to the additional space created at the front of the pocket.

The distributions of these different conformations across the different methods can be seen in Figure 7, where two distances between the oxygen atom of the ligand and residues Phe38 (backbone carbonyl oxygen atom) and Tyr120 (hydroxy oxygen atom) are used to describe the conformational space of the ligand. It is clear that while the ensembles generated by GCMC/MD, water hopping, and MD are similar, the GCNCMC/MD ensembles are distinct.

Analysis of the GCNCMC/MD nonequilibrium trajectories showed that the dry conformation was proposed only when the hydroxy group of the ligand moved across to occupy the crystal water site as the water was being decoupled. The synergistic nature of the GCNCMC move is therefore critical in generating this conformation and explains why it is not observed in GCMC/MD. The water-hopping method should theoretically also be able to generate these dry states. However, their absence can likely be attributed to the slower convergence time of this method, owing to the requirement of this method to sample from a much larger system volume.

The ligand conformation associated with the first wet state is observed to some extent by all four methods, as shown in Figure 7, although this is often for very short periods of time before returning to the crystal conformation. However, owing to the more efficient water sampling of GCNCMC/MD compared to the other methods, a water molecule is more frequently inserted onto the site shown by the black circle in Figure 6 (wet state 1) before the ligand flips back, stabilizing the conformation and prolonging its lifetime. The same process applies to the second wet state. Typically beginning from the first wet state, the ligand moves further to the back of the pocket, from where it either returns to the first wet state or a third water is inserted to stabilize this conformation, creating sufficient space for up to five waters within the binding site.

To ensure that the binding poses observed with GCNCMC/MD and not with the other methods were genuine, a representative simulation frame was taken from each of the four conformations shown in Figure 6 and simulations run with positional restraints applied to the protein and ligand heavy atoms. With the effect of ligand and protein conformational changes now removed, all methods should produce the same hydration networks. A detailed discussion of these simulations can be found in the Supporting Information. The results, shown in Figure S4, demonstrate that there is good agreement between the grand canonical methods, with both GCNCMD/MD and GCMC/MD predicting almost identical water locations and occupancies for the four binding poses. This suggests that these novel configurations are valid and are truly the result of GCNCMC/MD facilitating the configurational sampling of the ligand.

Simulations were also carried out to compare the methods’ abilities to produce converged results quickly and to equilibrate a water network in an initially dry binding site. The results are shown in Figures S5 and S6. While GCMC/MD appears to converge quickly in the unrestrained simulations, it is in fact showing false convergence. GCNCMC/MD, on the other hand, though appearing to converge more slowly, is tending toward a more reliable hydration state. Both GCMC/MD and GCNCNC/MD were similarly efficient at inserting waters into a dry binding site, as shown by Figure S7, with on the order of 10^5^ force evaluations being required. The water-hopping method was unable to produce converged results in the simulation times used and required approximately 1–2 orders of magnitude more force evaluations to equilibrate the water networks. Details of these simulations are provided in the Supporting Information.

Finally, to ensure that there was no dependence of the distributions being sampled on either the switching time of the move or the choice of the *n*_prop_ parameter, we performed simulations across a range of switching times, using a representative simulation frame of the MUP-I wet state 1 conformation, with position restraints applied. The results confirmed that there was no dependence of the distributions on either of these parameters. Details of these simulations, and their results, are reported in the Supporting Information and Figures S8 and S9.

## 5 Conclusions

Here, we have presented our implementation of NCMC-enhanced GCMC moves for the sampling of buried waters within the *grand* module,^33^ which we refer to in this work as GCNCMC/MD.

We compared the GCNCMC/MD method to conventional MD, GCMC/MD, and the recently published water-hopping method (as implemented in the *BLUES* package for *OpenMM*(49)). Results show that our GCNCMC/MD method can significantly enhance the sampling of bound waters, compared to existing methods. Not only is the efficiency on par with, or better than, current methods, but the ability to generate ligand conformations that were previously inaccessible demonstrates the efficacy of the technique in an unexpected fashion. Through the gradual development of the move proposal, the system was able to explore novel ligand configurations not observed in other methods.

It should be noted that since the completion of this work, other methods similar to the water-hopping method described here have been further developed (using parallelization across multiple GPUs) such that they may be more efficient than the GCNCMC/MD implementation in *grand*.^72^ Nonetheless, these methods lack one of the key advantages of grand canonical methods—their ability to tune the excess chemical potential of the solvent. This allows titrations to be performed across a range of chemical potential values, providing information on the relative thermodynamics of hydration sites within the system.^27^

The GCNMC/MD method was validated by comparing both the locations and occupancies of bound waters within three protein test systems with results obtained from the previously used GCMC/MD method: HSP90, trypsin, and MUP-I. We also demonstrate the ability of GCNCMC/MD to reproduce the density of bulk water obtained by MD sampling of the NPT ensemble, as shown in Figure S3.

The results presented highlight the impact that the water sampling can have on the ensembles generated through simulations performed on the MUP-I system. The configurations sampled by the GCNCMC/MD method were noticeably different from those sampled by MD, GCMC/MD, and water hopping as a result of both the increased efficiency and the gradual nature with which the trial states were generated. Given that the crystal structure contains only one water within the binding site, and the system is often used as a negative control, it was unexpected that increasing the degree of water sampling would have such a benefit for simulating the MUP-I system.

The dry state of the MUP-I system identified by the GCNCMC/MD simulation is a clear example of the method’s ability to drive the relaxation of orthogonal degrees of freedom through simply enhancing the water sampling. This binding pose was not observed by any of the other methods—either due to a lack of ligand relaxation during the deletion of the water, in the case of GCMC/MD, or due to the method being less efficient, where the chance of attempting a deletion move of the bound water is diminished, in the case of water hopping. We consider the ability of the GCNCMC/MD sampling to identify this dry state as a significant advantage.

We also tested and compared the efficiency of GCNCMC/MD with respect to the equilibration of water networks when starting from a dry binding pocket. We found that while the two grand canonical methods were comparable in terms of the number of force evaluations required to reach the equilibrium conformation, in both cases they were more efficient than the water-hopping method, by around 1–2 orders of magnitude.

Although not explored in detail here, the nonequilibrium work can be used to calculate the free energy of insertion/deletion of waters at certain points in space, using nonequilibrium free energy estimators.^73^ This has potential application for hydration sites within protein binding sites as it gives another quantitative measure of water-binding affinity, along with the occupancy. Future work will involve using the GCNCMC/MD method to generate a hydration free energy map of a binding site, through the values of the work obtained by attempting insertions and deletions at points throughout the pocket.

To conclude, the sampling of bound waters in protein–ligand systems is of huge importance in the context of free energy calculations. Despite this, conventional molecular dynamics is poor at effectively sampling bound waters, given the current available hardware. While grand canonical methods have been previously shown to improve the sampling of waters,^33^ our GCNCMC/MD implementation has been demonstrated to sample them more effectively and more efficiently, with the knock-on effect that the sampling of orthogonal degrees of freedom in the protein–ligand binding site is also improved. This has the potential in the future to improve the accuracy of binding free energy calculations where bound waters are present in the binding site.

## Data Availability

Scripts and input files used to generate the data shown throughout this work can be found at https://github.com/essex-lab/gcncmc-paper.

## Supporting Information Available

Full derivation of acceptance criteria; details and discussion of restrained simulations on the MUP-I system; details and discussion of simulations used to assess the convergence and efficiency of the different water-sampling methods; table of all NCMC protocols used; additional figures for simulation results not reported in the main text (PDF)
