# Supplementary material for: Enhanced Grand Canonical Sampling of Occluded Water Sites Using Nonequilibrium Candidate Monte Carlo
Source: J Chem Theory Comput. 2023 Jan 24;19(3):1050–62. doi: 10.1021/acs.jctc.2c00823 (PMC9933432; doi:10.1021/acs.jctc.2c00823)
Supplement: Supplementary file 1 — ct2c00823_si_001.pdf [file ct2c00823_si_001.pdf]

Supporting Information: Enhanced Grand Canonical Sampling of Occluded Water Sites Using Nonequilibrium Candidate Monte Carlo

Oliver J. Melling,[†] Marley L. Samways,[†] Yunhui Ge,[‡] David L. Mobley,[‡,¶] and Jonathan W. Essex[*,†]

†*School of Chemistry, University of Southampton, Southampton, SO17 1BJ, UK*

‡*Department of Pharmaceutical Sciences, University of California, Irvine, CA 92697, USA*

¶*Department of Chemistry, University of California, Irvine, CA 92697, USA*

E-mail: j.w.essex@soton.ac.uk

Derivation of Acceptance Criteria

The particle insertion and deletion moves used in a GCMC simulation make use of an ideal gas reservoir, from which the inserted particles are supplied. For the purposes of this derivation, we consider the simulated system and the ideal gas reservoir as one large, canonical system, where particles can only move between the ideal gas and system by GCMC moves.[S1] This ensemble contains a total of M particles, in a total volume of V (the system and ideal gas have volumes of V_{sys} and V_{ideal}, respectively), at a constant temperature of T, where the equilibrium probability for a configuration where N particles are found in the simulated

system is given by:

$$\pi\left(\mathbf{r}^N, \mathbf{r}^{M-N}, \mathbf{p}^M\right) = Q_{MVT}^{-1} h^{-3M} e^{-\beta E\left(\mathbf{r}^N, \mathbf{r}^{M-N}\right)} \mathrm{d}\mathbf{r}^M \mathrm{d}\mathbf{p}^M \tag{1}$$

where $\mathbf{r}^N$ represents the positions of the system particles, $\mathbf{r}^{M-N}$ represents those of the ideal gas, $\mathbf{p}^M$ are the momenta of all particles (note that these need not be separated), h is Planck's constant, Q_{MVT} is the partition function of this canonical ensemble, and E is the total energy, calculated as follows:

$$E(\mathbf{r}^N, \mathbf{r}^{M-N}, \mathbf{p}^M) = U(\mathbf{r}^N) + \sum_{i=1}^{M} \frac{|\mathbf{p_i}|^2}{2m} \tag{2}$$

Note that the potential energy has no dependence on the positions of the particles in the ideal gas.

We begin this derivation using the generalised acceptance criterion for moves employing nonequilibrium candidate Monte Carlo (NCMC), as published by Nilmeier et al.:[S2]

$$\frac{A(X|\Lambda_p)}{A(\tilde{X}|\tilde{\Lambda}_p)} = \frac{\pi(\widetilde{x}_T)}{\pi(x_0)} \frac{P(\tilde{\Lambda}_p|\widetilde{x}_T)}{P(\Lambda_p|x_0)} \frac{\alpha(\tilde{X}|\tilde{\Lambda}_p)}{\alpha(X|\Lambda_p)} e^{-\Delta\mathcal{S}(X|\Lambda_p)} \tag{3}$$

where $\pi(x_0)$ is the equilibrium probability of state x_0, $P(\Lambda_p|x_0)$ is the probability of selecting protocol Λ_p, given state x_0, $\alpha(X|\Lambda_p)$ is the cumulative probability of all the perturbation steps from protocol Λ_p, and $\Delta\mathcal{S}(X|\Lambda_p)$ is the conditional path action difference. The ratio of equilibrium probabilities is simply dependent on the exponentiated change in total energy over the course of the move:

$$\frac{\pi(\widetilde{x}_T)}{\pi(x_0)} = e^{-\beta\Delta E(X|\Lambda_p)} \tag{4}$$

In the *grand* module, a single type of nonequilibrium protocol is defined per move, where the direction in which this protocol is applied is determined by whether the particle is alchemically coupled or decoupled (or: inserted or deleted). Therefore, the cumulative probability of the perturbation steps being applied is equal for insertion and deletion moves, so that

$\alpha(X|\Lambda_p) = \alpha(\tilde{X}|\tilde{\Lambda}_p)$, and therefore:

$$\frac{\alpha(\tilde{X}|\tilde{\Lambda}_p)}{\alpha(X|\Lambda_p)} = 1 \tag{5}$$

More important here, is the probability of proposing the forward and reverse moves, denoted by $P(\Lambda_p|x_0)$ and $P(\tilde{\Lambda}_p|\tilde{x}_T)$, respectively. An insertion move involves choosing a particle at random from the ideal gas, and inserting it at a random location (with infinitesimal volume, d$\mathbf{r}$) in the simulated system, whilst retaining its orientation — in practice, this involves generating a random orientation upon insertion, to account for the fact that the ideal gas is not explicitly simulated. The probability of proposing such a move is:

$$P(\Lambda_p|x_0) = \frac{1}{M-N}\frac{\mathrm{d}\mathbf{r}}{V_{sys}} \tag{6}$$

where the first term is the probability of selecting a specific ideal gas particle at random, and the second is the probability of choosing a particular insertion point at random. Conversely, the probability of attempting a deletion move to return the particle to the same location in the ideal gas is:

$$P(\tilde{\Lambda}_p|\tilde{x}_T) = \frac{1}{N+1}\frac{\mathrm{d}\mathbf{r}}{V_{ideal}} \tag{7}$$

where it should be noted that the first term accounts for the fact that the simulated system contains $N+1$ particles, following an insertion. The ratio of these two terms is:

$$\frac{P(\tilde{\Lambda}_p|\tilde{x}_T)}{P(\Lambda_p|x_0)} = \frac{M-N}{V_{ideal}}\frac{V_{sys}}{N+1} \tag{8}$$

For a system of N particles in a volume V, the ideal component of the canonical partition function can be calculated analytically by:

$$Q_{NVT}^{id} = \frac{V^N}{\Lambda^{3N}N!} \tag{9}$$

where Λ is the thermodynamic wavelength. From Eq. 9 the ideal contribution to the Helmholtz free energy for the system particles can also be calculated analytically:

$$F^{id}(N) = -k_B T \ln\left(\frac{V^N}{\Lambda^{3N} N!}\right) \tag{10}$$

Assuming a very large ideal gas allows the use of Stirling's approximation, applicable for very large numbers, such that Eq. 10 can be rewritten as:

$$F^{id}(N) \approx -k_B T \left(N \ln\left(\frac{V}{\Lambda^3}\right) - N \ln N + N\right) \tag{11}$$

The chemical potential is defined as the derivative of the free energy with respect to the particle number meaning the ideal chemical potential can be calculated as:

$$\mu^{id} = \frac{\partial F^{id}}{\partial N} = -k_B T \ln\left(\frac{V}{N\Lambda^3}\right) \tag{12}$$

This allows the chemical potential to be related to the number density of the ideal gas (ρ_{ideal}) as follows:

$$\mu = k_B T \ln(\rho_{ideal}\Lambda^3) \tag{13}$$

In the limit of an infinitely large ideal gas, the first term of Eq. 8 reduces to the number density of the ideal gas meaning the relationship in Eq. 13 allows the probability ratio in Eq. 8 to be written as:

$$\frac{P(\tilde{\Lambda}_p|\tilde{x}_T)}{P(\Lambda_p|x_0)} = \frac{e^{\beta\mu}}{\Lambda^3}\frac{V_{sys}}{N+1} \tag{14}$$

Eqs. 3, 4, 5 and 14 can then be combined to give:

$$\frac{A(X|\Lambda_p)}{A(\tilde{X}|\tilde{\Lambda}_p)} = \frac{1}{N+1}\frac{V_{sys}}{\Lambda^3} e^{\beta\mu} e^{-\beta\Delta E(X|\Lambda_p)} e^{-\Delta\mathcal{S}(X|\Lambda_p)} \tag{15}$$

The Adams parameter[S3,S4] is defined as:

$$B = \beta\mu + \ln\left(\frac{V_{sys}}{\Lambda^3}\right) \tag{16}$$

Substituting the Adams parameter into Eq. 15 gives:

$$\frac{A(X|\Lambda_p)}{A(\tilde{X}|\tilde{\Lambda}_p)} = \frac{1}{N+1}e^{B}e^{-\beta\Delta E(X|\Lambda_p)}e^{-\Delta\mathcal{S}(X|\Lambda_p)} \tag{17}$$

When using equilibrium-preserving propagation techniques (such as the BAOAB Langevin integrator used in this work[S5,S6]), it has been shown that the conditional path action difference is related to the negative heat of the nonequilibrium process:[S2]

$$\Delta\mathcal{S}(X|\Lambda_p) = -\beta Q(X|\Lambda_p) \tag{18}$$

THe heat can be calculated as the sum of the total energy change of the system over all the propagation steps:

$$Q(X|\Lambda_p) = \sum_{t=1}^{T}\left[E(x_t) - E(x_t^*)\right] \tag{19}$$

where the sum runs over all the propagation kernels, with $E(x_t^*)$ and $E(x_t)$ representing the energy of the system before and after relaxation.

The energy change of the system over the course of the move can be expressed in terms of heat and work:

$$\Delta E(X|\Lambda_p) = W(X|\Lambda_p) + Q(X|\Lambda_p) \tag{20}$$

The acceptance ratio in Eq. 17 can therefore be rewritten in terms of the nonequilibrium work, $W(X|\Lambda_p)$:

$$\frac{A(X|\Lambda_p)}{A(\tilde{X}|\tilde{\Lambda}_p)} = \frac{1}{N+1}e^{B}e^{-\beta(W(X|\Lambda_p)+Q(X|\Lambda_p))}e^{\beta Q(X|\Lambda_p)} = \frac{1}{N+1}e^{B}e^{-\beta W(X|\Lambda_p)} \tag{21}$$

The corresponding derivation for a deletion move follows the same steps, and arrives at

the following acceptance ratio:

$$\frac{A(X|\Lambda_p)}{A(\tilde{X}|\tilde{\Lambda}_p)} = Ne^{-B}e^{-\beta W(X|\Lambda_p)} \tag{22}$$

It should be noted that Eqs. 21 and 22 very closely resemble those of instantaneous GCMC moves, with the only difference being the replacement of the potential energy change, with the nonequilibrium work.

When running a simulation in which the GCMC region is a sphere, focused on a particular region, V_{sys} must be replaced with the volume of the GCMC sphere. Additionally, the value of N must correspond to the number of water molecules within the GCMC sphere — given that *grand* allows waters to permeate the GCMC region, this must be updated at the end of each move, in case the number of water molecules has changed by more than one. Therefore, Eqs. 21 and 22 are replaced with Eqs. 23 and 24:

$$\frac{A(X|\Lambda_p)}{A(\tilde{X}|\tilde{\Lambda}_p)} = \frac{1}{N_T}e^{B}e^{-\beta W(X|\Lambda_p)} \tag{23}$$

$$\frac{A(X|\Lambda_p)}{A(\tilde{X}|\tilde{\Lambda}_p)} = N_0 \ e^{-B}e^{-\beta W(X|\Lambda_p)} \tag{24}$$

where N_0 is the number of waters present in the sphere in the initial state (x_0) and N_T is the number present in the sphere in the proposed state ($\tilde{x}_T$). If the water which is subjected to the switching ends up outside the GCMC sphere, the move must be automatically rejected as the reverse move is impossible, as the probability of the reverse protocol becomes zero.

Restrained Simulations To further ensure that the GCNCMC/MD method could predict both water locations and their occupancies with similar results to GCMC/MD, we selected a representative state from each of the four binding poses, applied restraints to the non-solvent heavy atoms and performed simulations with each of GCNCMC/MD, GCMC/MD and water hopping as described previously. This provides a better comparison between the methods, as it removes the issue of the different protein-ligand sampling observed, thereby reducing the comparison to just the sampling of water binding sites. We therefore expect to see much better agreement between the different methods for the number of waters observed in the binding site. The results are shown in Fig. S4.

For the dry state, GCNCMC/MD and GCMC/MD generated 98.6 $\pm$ 0.1 % and 98.5 $\pm$ 0.1 % of frames respectively with no waters present in the binding region. The remaining frames contained either one or two transient waters which had an even spatial distribution about the binding region and none of which persisted for longer than a few simulation frames. One of the eight water hopping repeats inserted a water into the binding region. This water was subsequently deleted after 1123 moves, compared to GCNCMC/MD where nearly all waters inserted were deleted within 10 moves of their insertion.

Similar results are observed for the crystal state, with the grand canonical methods showing populations of 99.2 $\pm$ 0.1 % and 99.1 $\pm$ 0.1 % for the one-water conformation, with the remaining states containing a second water. At no point was the crystal water deleted. Water hopping did not accept any moves resulting in translation into or out of the binding region.

Wet state 1 again showed strong agreement between the GCNCMC/MD and GCMC/MD results with the population of the states containing 1-4 waters being almost identical across the two methods. As expected, the majority of states contained the two waters shown in Fig. 6 in the main text with the remaining states containing either 1, 3 or 4 waters. The slightly greater variance in the data for this state compared to the previous two can be attributed to the increased space created by the ligand conformation, increasing the acceptance probabil-

ities of insertion moves. The slow convergence times of water hopping were highlighted by only four of the 8 repeats inserting a third water. Whereas the GCNCMC/MD simulations led to a water binding/unbinding event on average once every 100 moves, the water hopping simulations only achieved this on average once every 5333 moves.

Wet state 2 saw small differences between the two grand canonical methods. This is, again, owing to the additional space created by the ligand moving to the back of the pocket allowing for greater variation in the number of waters present — the water sites also appear to be less well defined, as more space is available. The starting structure contained five waters, as shown in Fig. 6 in the main text, and the results show that 3, 4 or 5 waters within the binding site are all reasonably populated configurations. Both GCNCMC/MD and GCMC/MD generated a few states with only 1 or 2 waters and one 6-water state was accepted with GCNCMC/MD, lasting 3 moves before a water was deleted. In 6 out of the 8 repeats using the water hopping method, the waters inside the pocket were left unchanged. In the other two repeats, a water was successfully translated out of the binding region which, in one case, was subsequently replaced by the translation of another water. Across the 8 repeats of each method, GCMC/MD simulations accepted on average one move every $(3.5 \pm 0.7) \times 10^5$ force evaluations, GCNCMC/MD every $(6.4 \pm 0.4) \times 10^5$ force evaluations and water hopping one every $(7.1 \pm 5.0) \times 10^6$ force evaluations.

Comparison of Simulation Convergence and Efficiency A qualitative comparison of the number of moves required for a simulation to reach convergence was also performed. This was done by plotting the average number of waters within the binding site over the course of each individual simulation performed on the unrestrained MUP-I and the MUP-I system restrained in the four conformations as described in the main text. The results are shown in Figs. S5 and S6 with both the number of MC moves and the number of force evaluations being used as metrics to quantify efficiency. Drawing comparisons between GCMC/MD and the two NCMC-based methods is not straightforward when considering the number of MC moves required, given the different nature of the moves. Using force

evaluations however (Fig. S6), shows that GCMC/MD appears to be the quickest to produce converged results, particularly for the unrestrained simulations. It is important to consider though that this is partly owing to the method's inability to capture some of the ligand conformations observed with GCNCMC/MD and therefore although the results may appear converged, they have not necessarily converged to the correct value. The water hopping method clearly does not converge within the simulation time, with only a few moves being accepted across the eight repeats performed.

Simulations were also run to assess the efficiency with which the different methods could equilibrate the water network in a dry binding site. Here, we use the crystal state and wet state 1 of MUP-I to assess the ability of GCNCMC/MD, GCMC/MD and water hopping to equilibrate the number of waters within the cavity — in each case starting with no waters in the binding site. For the sake of this investigation, we define the system to be equilibrated at the first point that there is a water molecule within 1.4 Åof the hydration site(s) previously identified as shown in Fig. 6 in the main text (one site for the crystal state and two sites for wet state 1). Restraints were applied as before to prevent protein and ligand conformational changes previously observed.

For the two NCMC methods, simulations were run over a range of switching times (n_{prop}=50) and moves were separated by 1000 steps of MD (2 ps). The GCMC/MD simulations were run in iterations of 50 GCMC moves followed by 1000 steps MD.

As shown in Fig. S7, on average GCNCMC/MD and GCMC/MD require a similar number of force evaluations to equilibrate the waters within the binding site, with the results appearing fairly independent of switching time. Water hopping takes longer than both grand canonical methods to achieve the same equilibration. To equilibrate the crystal state using GCNCMC/MD, an average of $(1.8 \pm 0.3) \times 10^5$ force evaluations were required, using GCMC/MD required $(2.5 \pm 1.1) \times 10^5$ and water hopping $(5.6 \pm 0.9) \times 10^6$ force evaluations.

For the wet state of MUP-I tested, the GCMC/MD sampling proved the most efficient, requiring an average of $(1.3 \pm 0.3) \times 10^5$ force evaluations and GCNCMC/MD an average

of $(2.6 \pm 0.3) \times 10^5$. The gap between the grand canonical methods and water hopping is greater, with the latter taking an average of $(3.8 \pm 0.5) \times 10^7$ force evaluations to equilibrate the two waters. In general, GCMC/MD is the most efficient method for equilibrating water networks in a dry binding pocket, although this relies on the assumption that there are no coupled protein or ligand motions, necessary for the equilibration of the water network, that GCMC/MD would fail to sample.

Dependence of Sampled Distributions on Switching Time and n_{prop} To confirm that the choice of both the switching time, and the n_{prop} parameter has no effect on the distributions being sampled we performed additional simulations across a range of switching times and n_{prop} values. We selected a representative frame of the MUP-I system in the wet state 1 conformation, and applied positional restraints to the protein and ligand heavy atoms with a force constant of 10 kcal Å^{-2} mol^{-1}. This ensured that there was limited sampling of the protein and ligand motions that might otherwise cause differences in the ensembles generated.

To test the switching times, values of 5, 7, 9, 11, 13 and 15 ps were used with five independent repeats performed at each switching time. The results are shown in Fig. S8 where it can be seen that there is no discernible correlation between the switching time used and the number of waters observed.

To test the n_{prop} values, we used a constant switching time of 10 ps and n_{prop} values of 10, 20, 40, 50 and 100. The results are shown in Fig. S9 and again there is no correlation between the parameter choice and the observed water occupancy.

These results provide further evidence that the additional conformations we observe with the GCNCMC/MD method are in fact genuine and physically relevant conformations, and not a result of our choice of parameters.

NCMC Simulation Protocols

Table S1: Details of the simulation protocols used for GCNCMC/MD and water hopping simulations on the protein test systems. In the cases of the water hopping simulations for HSP90 and restrained MUP-I, the switching times used were double those used for GCNCMC/MD as water hopping involves both a decoupling and a subsequent coupling of a water whereas GCNCMC/MD only requires one or the other. The n_{pert} value refers to the the total number of perturbations per NCMC move.

System	Method	No. repeats	Switching Time / ps	nprop	npert
HSP90	GCNCMC/MD	3	5	20	124
HSP90	GCNCMC/MD	3	5	50	49
HSP90	GCNCMC/MD	3	10	20	249
HSP90	GCNCMC/MD	3	10	50	99
HSP90	Water Hopping	3	10	20	249
HSP90	Water Hopping	3	10	50	99
HSP90	Water Hopping	3	20	20	499
HSP90	Water Hopping	3	20	50	199
Trypsin	GCNCMC/MD	4	9	20	224
Trypsin	GCNCMC/MD	4	7	50	69
MUP-I (unrestrained)	GCNCMC/MD	3	7	20	174
MUP-I (unrestrained)	GCNCMC/MD	3	7	50	69
MUP-I (unrestrained)	GCNCMC/MD	3	9	20	224
MUP-I (unrestrained)	GCNCMC/MD	3	9	50	89
MUP-I (unrestrained)	Water Hopping	2	7	20	174
MUP-I (unrestrained)	Water Hopping	2	7	50	69
MUP-I (unrestrained)	Water Hopping	2	9	20	224
MUP-I (unrestrained)	Water Hopping	2	9	50	89
MUP-I (restrained)	GCNCMC/MD	All (32)	7	20	174
MUP-I (restrained)	Water Hopping	All (32)	14	20	349

Bulk Water Acceptance Rate and Efficiency

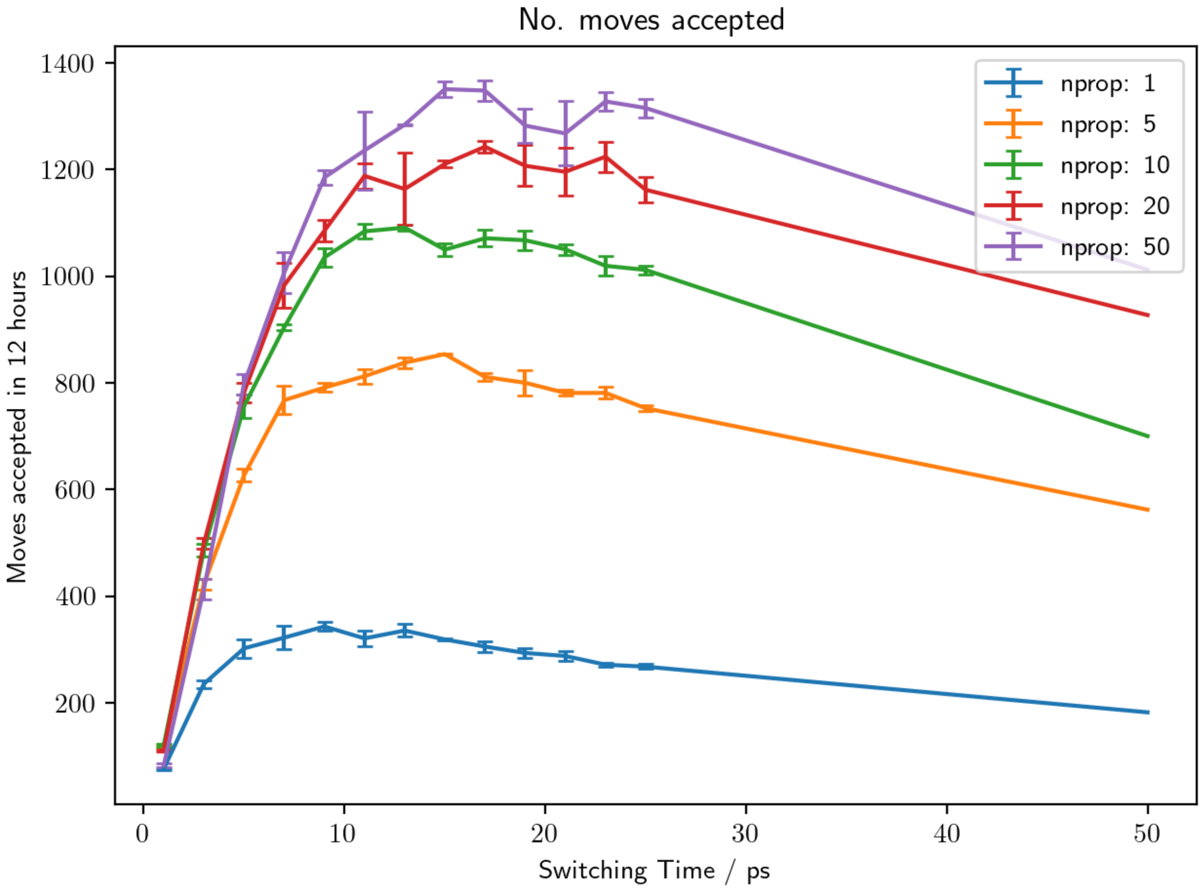

Figure S1: The efficiency of different GCNCMC protocols when carried out on a water box. The protocols are grouped by the n_{prop} parameter which defines the number of MD steps between each perturbation step during the GCNCMC move. Efficiency is defined as the number of accepted moves within 12 hours of wall time. Data points have been included at a switching time of 50 ps to highlight the decrease in efficiency after the broad peak.

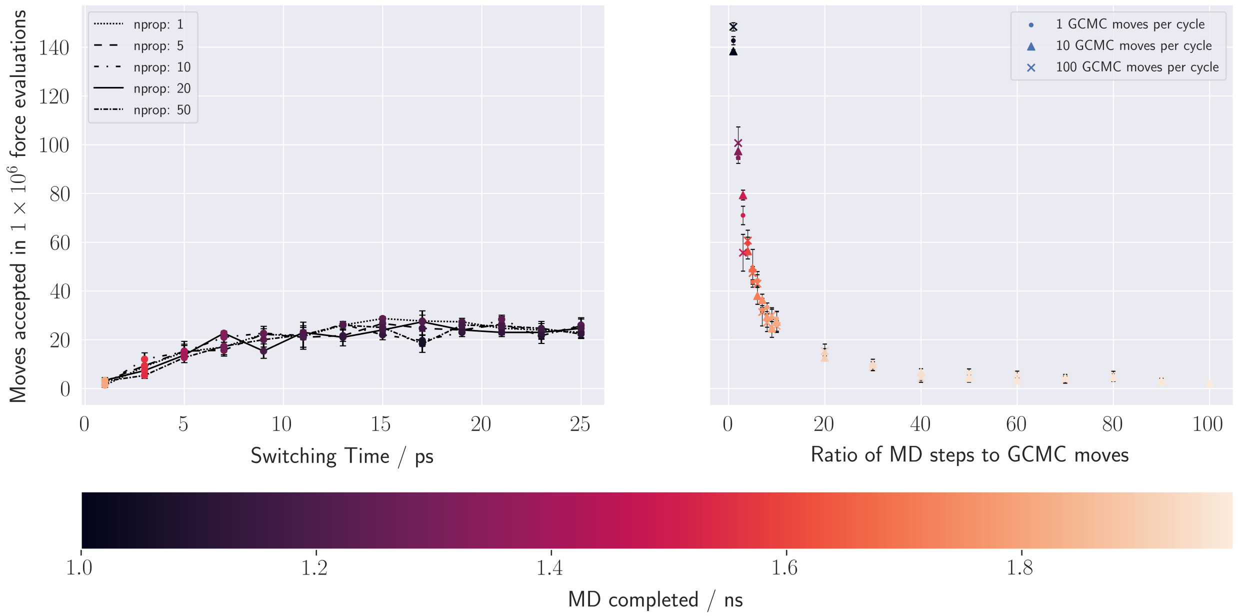

Figure S2: Moves accepted within 1×10^6 force evaluations for a range of GCNCMC/MD (left) protocols and GCMC/MD (right) protocols on a bulk water system. Data points are coloured based on the amount of MD sampling performed on the system. MD performed during accepted GCNCMC moves is included in this calculation. The GCNCMC/MD data are grouped based on the number of MD steps between NCMC moves and plotted against the switching time of a single move. The GCMC/MD data are grouped by the number of moves run per iteration and plotted against the ratio of the MD steps to GCMC moves. The same y-axis is used for both plots.

Bulk Water Densities

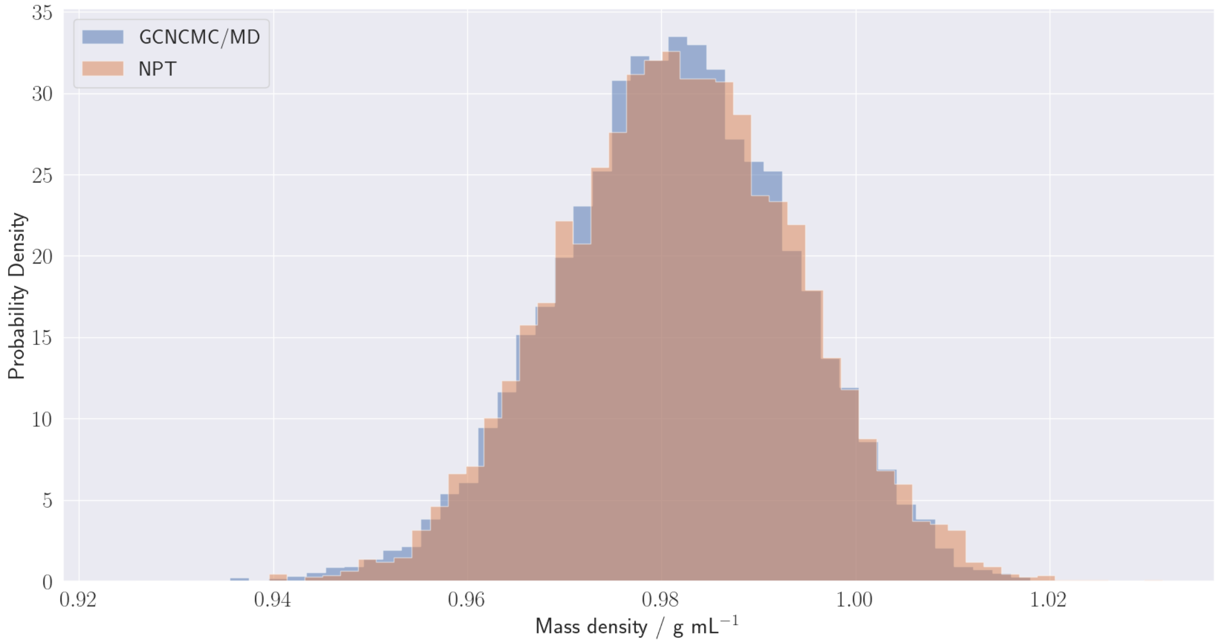

Figure S3: An equilibrated system containing 500 water molecules was used to ensure the GCNCMC/MD method could reproduce the same density as MD sampling from the NPT ensemble. Three 100 ns NPT simulations were performed with the density recorded every 50 ps. A Monte Carlo barostat was used to maintain the pressure at 1 bar by attempting volume changes every 25 time steps. Three GCNCMC/MD simulations were also run by setting the volume to the average value observed during the NPT simulations. A switching time of 7 ps was used, split into 69 perturbations — each separated by 50 steps of MD. Iterations consisted of a single GCNCMC move followed by 3 ps MD. A total of 150,000 iterations were performed for each repeat with the density recorded every 50 iterations.

Restrained Simulations

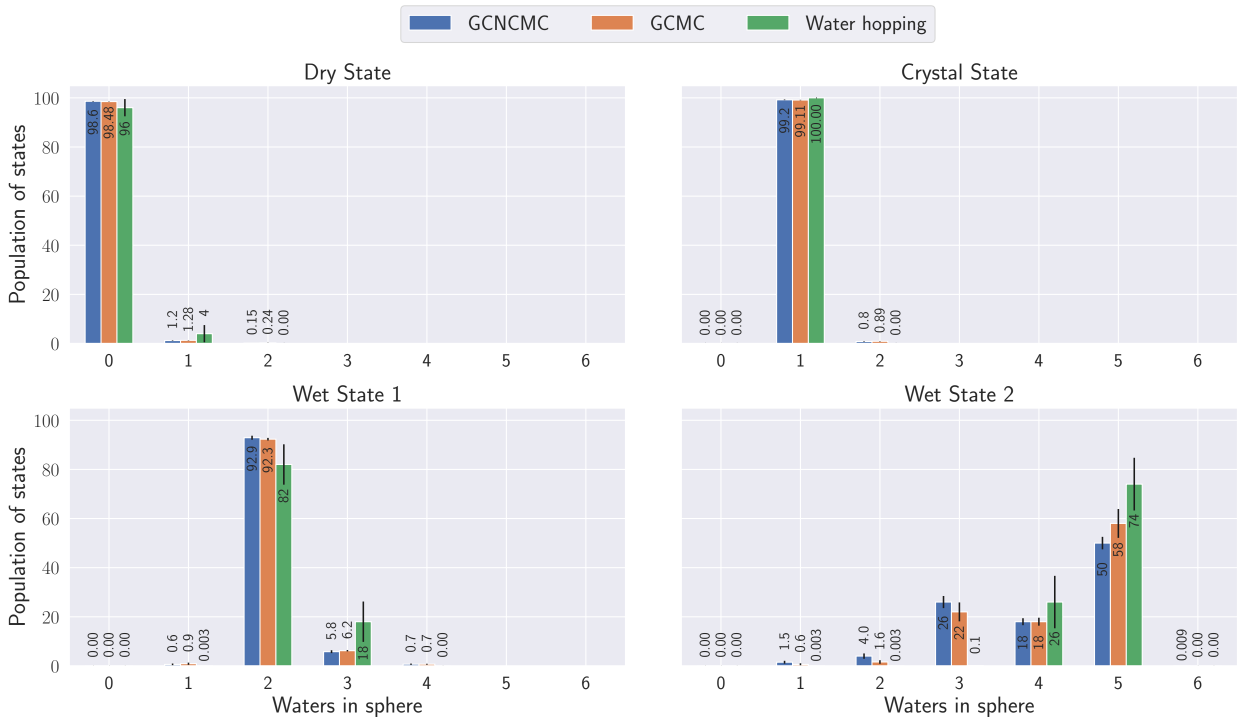

Figure S4: Distributions of the number of waters observed using each of the water placement methods in the MUP-I binding site, when the protein-ligand complex is restrained to the previously described conformations. Positional restraints were applied to a representative structure of each of the four dominant MUP-I conformations previously identified. Simulations were performed using the three different methods (GCNCMC/MD, GCMC/MD and water hopping) to assess the agreement and convergence of the results. Eight independent repeats were run for each combination of method and conformation. The number of waters present in the binding site is shown along the x-axis with the height of the bars indicating the average percentage of frames containing each number of waters. The error bars show the standard error of the mean

Simulation Convergence and Efficiency

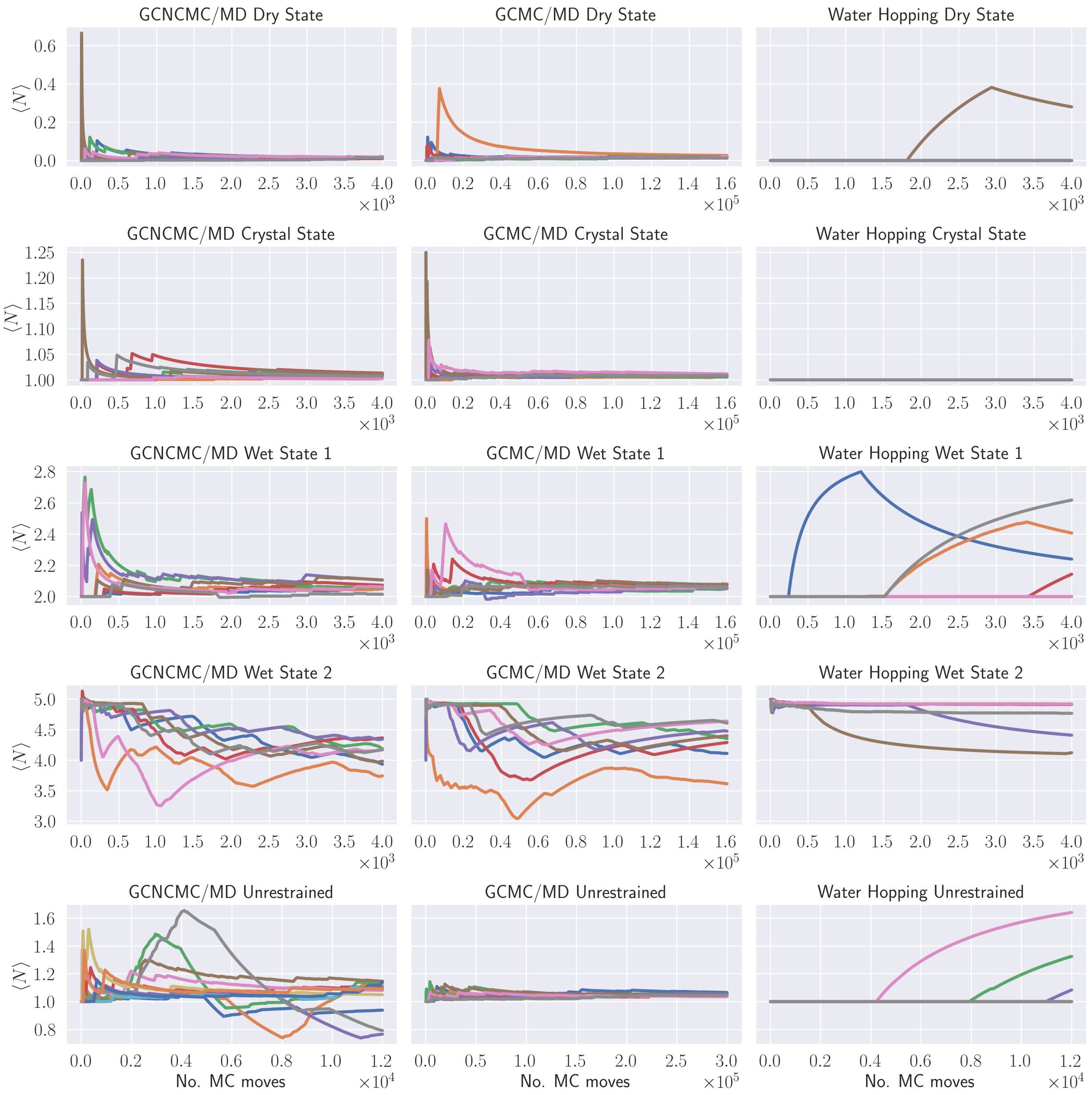

Figure S5: Convergence results from the restrained and unrestrained simulations performed on the four dominant conformations of MUP-I. The average number of waters within the GCMC sphere ($\langle N \rangle$) is plotted against the number of Monte Carlo moves performed for the eight different repeats run for each combination of method and MUP-I conformation. The rows show the dry state, crystal state, wet state 1, wet state 2 and unrestrained simulations respectively. The columns show the GCNCMC/MD, GCMC/MD and water hopping method.

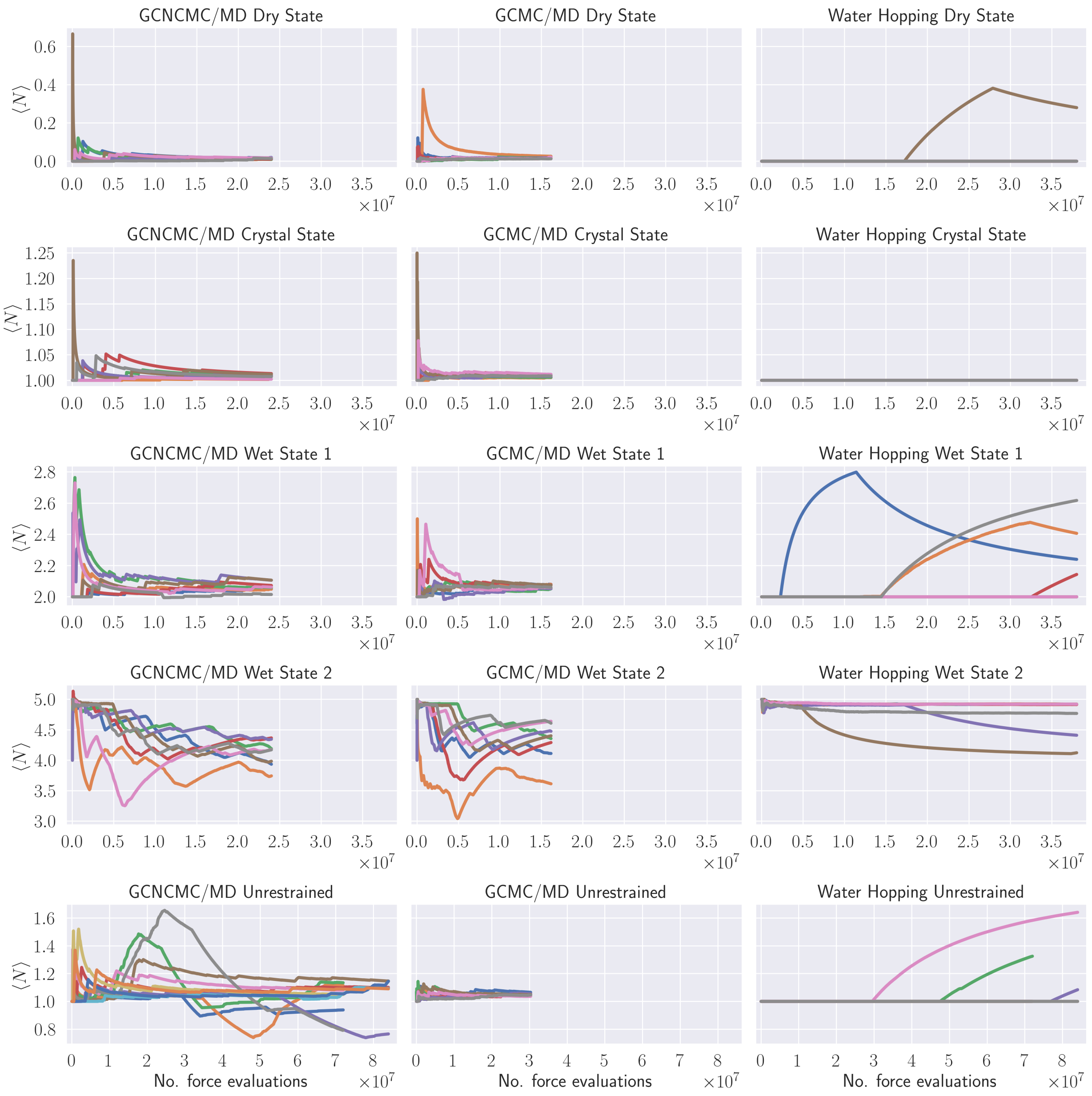

Figure S6: Convergence results from the restrained and unrestrained simulations performed on the four dominant conformations of MUP-I. The average number of waters within the GCMC sphere ($\langle N \rangle$) is plotted against the number of force evaluations required for the eight different repeats run for each combination of method and MUP-I conformation. The rows show the dry state, crystal state, wet state 1, wet state 2 and unrestrained simulations respectively. The columns show the GCNCMC/MD, GCMC/MD and water hopping method.

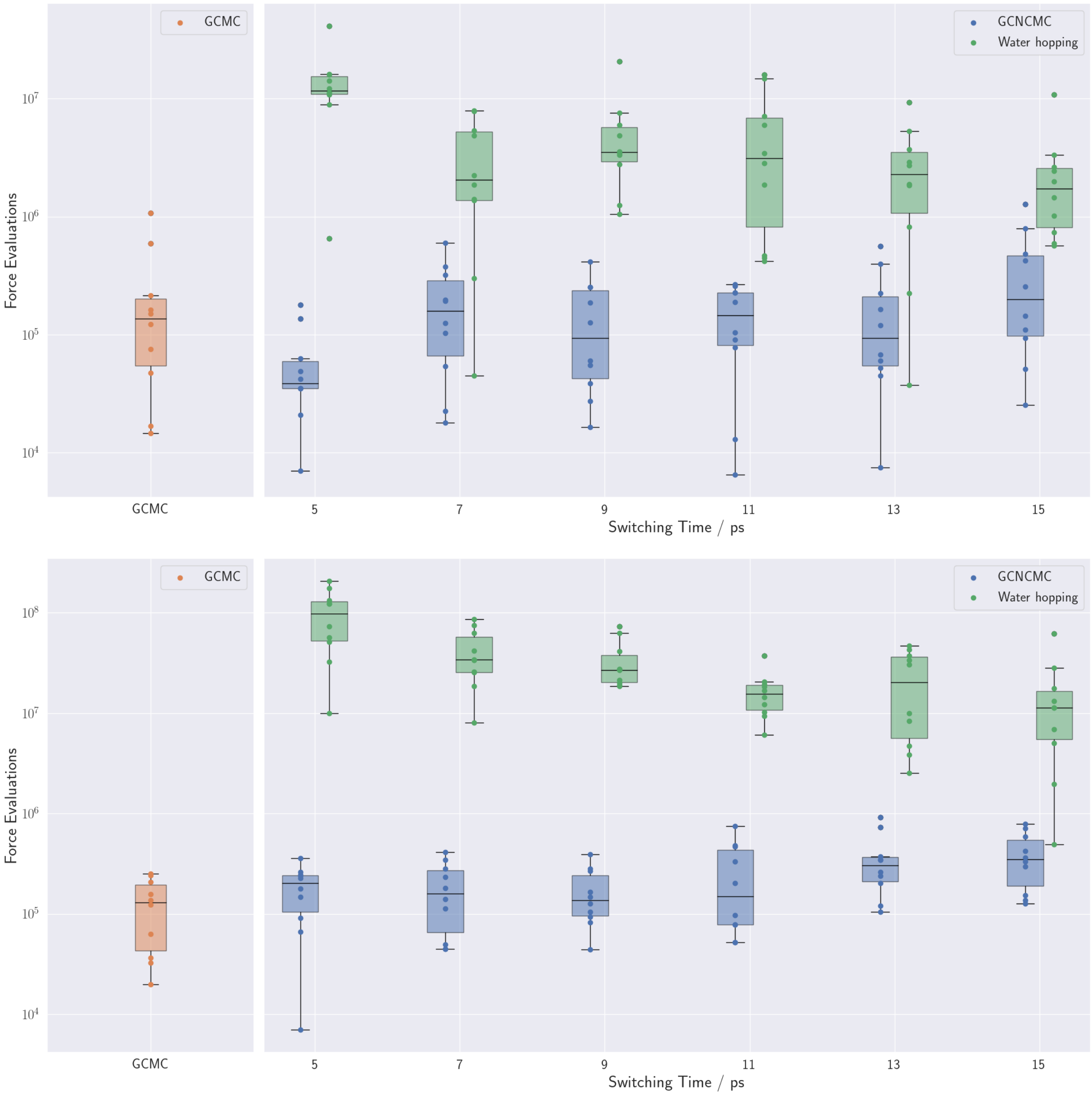

Figure S7: The number of force evaluations required to equilibrate the crystal state (top) and first wet state (bottom) of MUP-I shown for the three different methods starting from no waters present. Note that a log scale is used for the y-axis. The offset of the boxes is for ease of interpretation; the switching times used are labelled on the x-axis. The whiskers of the box plots extend to show the range of the data, with the exception of any outliers. The boxes show the upper and lower quartiles as well as the median.

Dependence on Switching Time

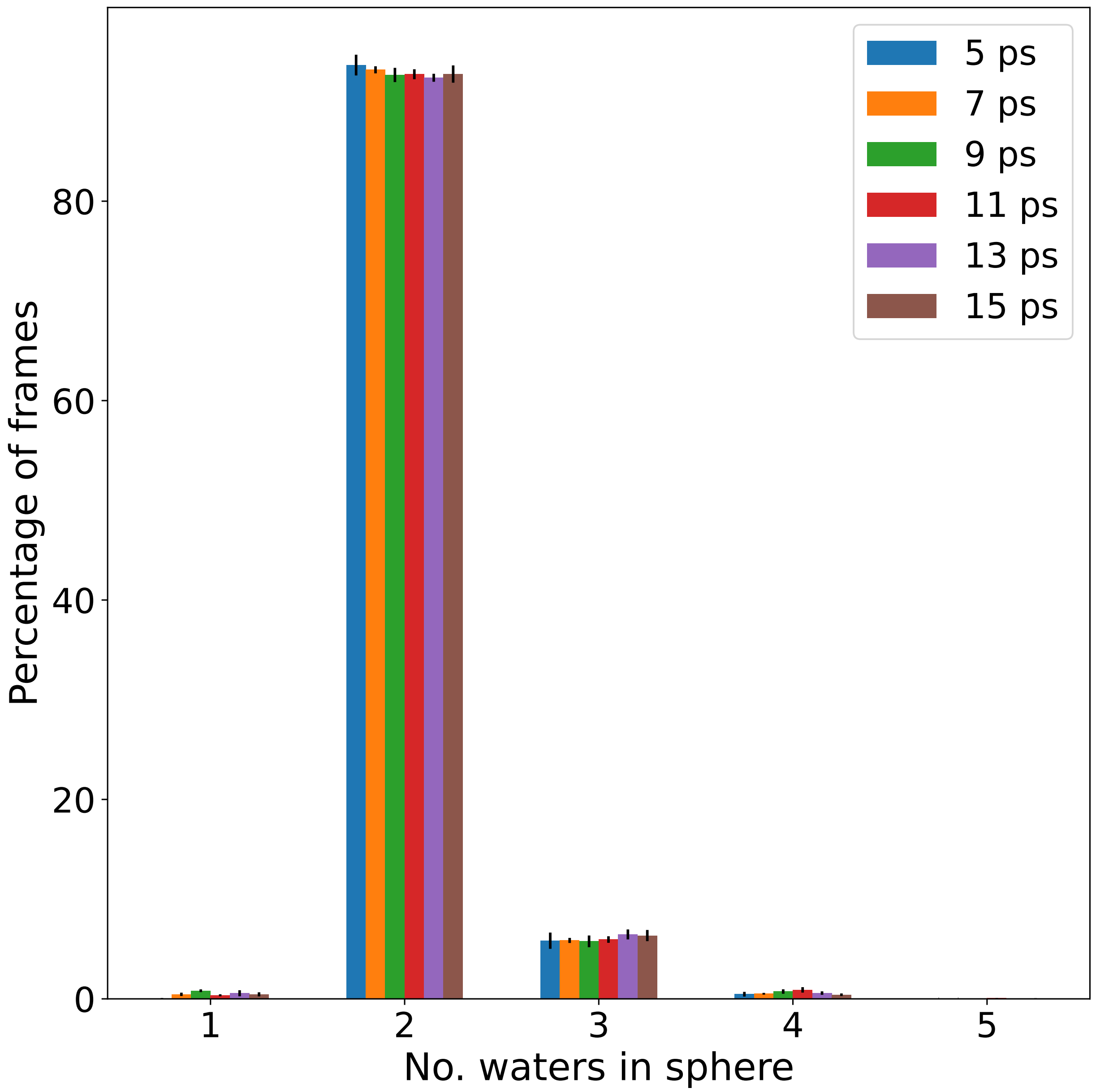

Figure S8: Distribution of the number of waters observed in the MUP-I binding site with the protein and ligand restrained to the wet state 1 conformation. Simulations were performed across 6 different switching times ranging from 5 to 15 ps, in increments of 2 ps. A fixed n_{prop} value of 50 was used for each switching time. The error bars show the standard error of the mean calculated across 5 repeats.

Dependence on nprop

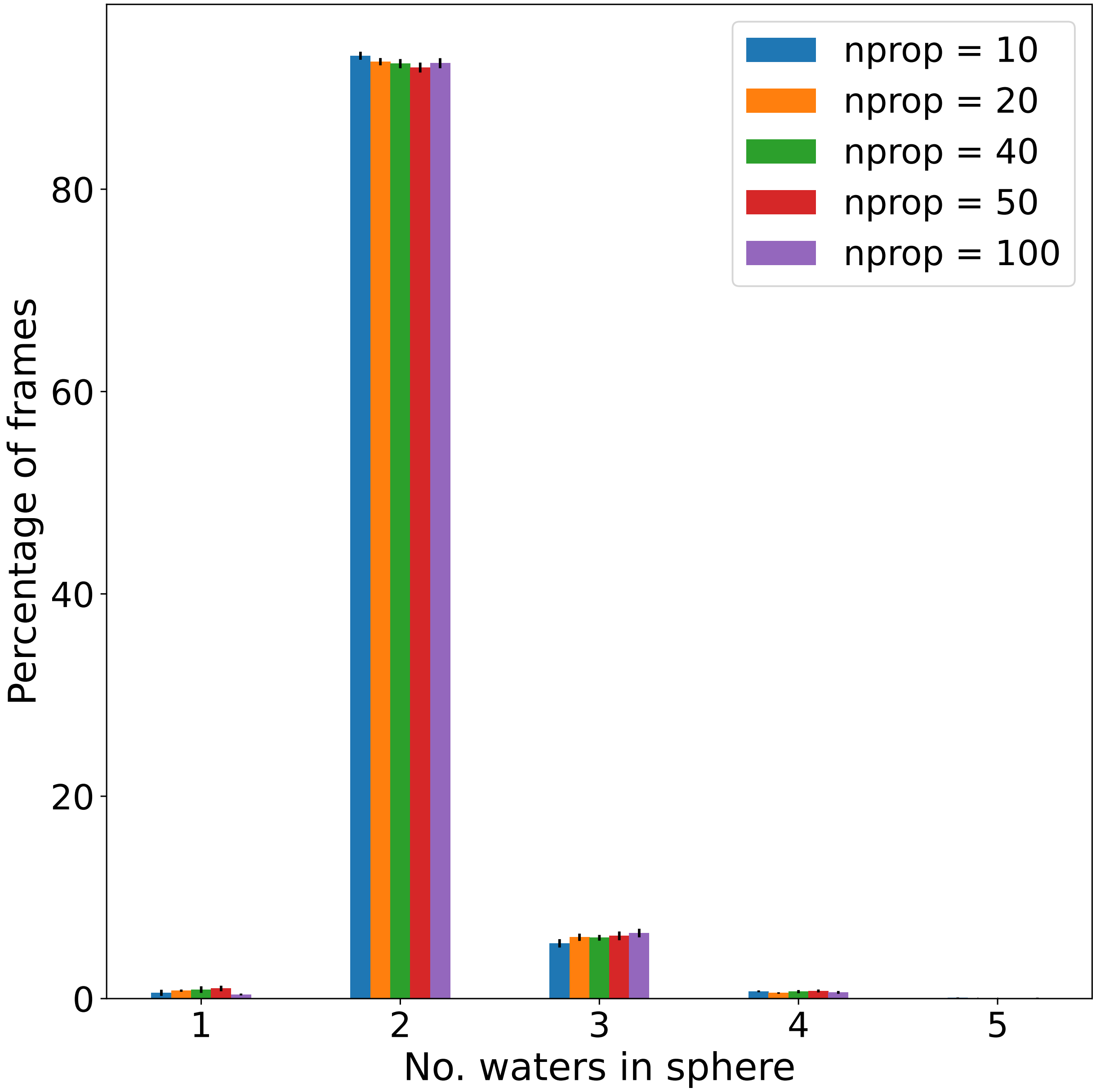

Figure S9: Distribution of the number of waters observed in the MUP-I binding site with the protein and ligand restrained to the wet state 1 conformation. Simulations were performed across 5 different n_{prop} values: 10, 20, 40, 50 and 100. A fixed switching time of 10 ps was used for each simulation. The error bars show the standard error of the mean calculated across 5 repeats.

References

(S1) Frenkel, D.; Smit, B. *Understanding molecular simulation: from algorithms to applications*, 2nd ed.; Computational science series 1; Academic Press: San Diego, 2002.

(S2) Nilmeier, J. P.; Crooks, G. E.; Minh, D. D. L.; Chodera, J. D. Nonequilibrium candidate Monte Carlo is an efficient tool for equilibrium simulation. *Proc. Natl. Acad. Sci. USA* **2011**, *108*, E1009–E1018.

(S3) Adams, D. Chemical potential of hard-sphere fluids by Monte Carlo methods. *Mol. Phys.* **1974**, *28*, 1241–1252.

(S4) Adams, D. Grand canonical ensemble Monte Carlo for a Lennard-Jones fluid. *Mol. Phys.* **1975**, *29*, 307–311.

(S5) Fass, J.; Sivak, D.; Crooks, G.; Beauchamp, K.; Leimkuhler, B.; Chodera, J. Quantifying Configuration-Sampling Error in Langevin Simulations of Complex Molecular Systems. *Entropy* **2018**, *20*, 318.

(S6) Leimkuhler, B.; Matthews, C. Rational Construction of Stochastic Numerical Methods for Molecular Sampling. *Appl. Math. Res. eXpress* **2012**, *34*, DOI: 10.1093/amrx/abs010.
